# Modeling of network public opinion communication based on social combustion theory under sudden social hot events

**DOI:** 10.1371/journal.pone.0311968

**Published:** 2024-11-06

**Authors:** Tinggui Chen, Tiantian Wu, Jianjun Yang, Beijia Xu

**Affiliations:** 1 School of Statistics and Mathematics, Zhejiang Gongshang University, Hangzhou, China; 2 Academy of Zhejiang Culture Industry Innovation & Development, Zhejiang Gongshang University, Hangzhou, China; 3 Department of Computer Science and Information Systems, University of North Georgia, Oakwood, Georgia, United States of America; 4 College of International Education, Zhejiang Gongshang University, Hangzhou, China; Bina Nusantara University, INDONESIA

## Abstract

Sudden social hot events spread rapidly in the Internet era, which can easily cause group behaviors. Therefore, studying the law of public opinion communication has important theoretical and practical significance for controlling public opinion. This paper constructs the DBUS (Divorced-Burning-Unburning-Stable) model according to the social combustion theory deduced from the analogy of natural combustion phenomena. This paper comprehensively considers the influence of individual characteristics and external environment on the dissemination of online public opinion, introduces indicators such as individuals’ self-awareness ability, authority to reflect individual differences, and takes the surrounding individual influence as an external environment factor. Through simulations, we find that (1) the speed of public opinion communication and the dissipation speed are related to cognitive ability, conformity and the network structure; (2) the credibility of the government and the media and the transparency of the information released are the most important factors affecting the scale of public opinion communication; In addition, the rationality and effectiveness of the model established in this paper are verified by an example.

## Section 1: Introduction

Sudden social hot events typically refer to events that occur abruptly within society, attracting widespread attention and discussion. These events often have urgency, uncertainty, and impact, and may significantly affect public safety, social order, the environment, or people’s psychology. In recent years, unexpected social hot events, such as the “Beating incident in a Tangshan BBQ restaurant”, the “Stampede accident in Itaewon, Seoul, South Korea” and the “Xiao Yangge’s public image collapse”, have constantly attracted people’s attention. With the help of the Internet, public opinion can easily induce extreme social emotions, trigger network mass incidents. Based on this, a network public opinion communication model is constructed which analyzes the real evolutionary process of public opinion. The ability of the emergency management department to carry out public opinion guidance and control has important practical significance.

At present, in the context of sudden social hot events, most of the research on network public opinion communication in academia relies on the characteristics of complex network structures and classic infectious disease models. Modern communication studies show that network public opinion communication involves incubation, diffusion, and recession, which are inherently similar to the occurrence mechanisms of combustion phenomena in nature. Therefore, the social combustion theory deduced by the analogy of combustion phenomena in nature is feasible to study the process of network public opinion communication. However, this theory is widely used in social stability early warning and regulation systems, emergency response model construction, and evolution analysis of mass events [1], and a few scholars have introduced it into the field of public opinion communication research. Li et al. [2] proposed using combustion theory in social networks to characterize the interaction between users. However, most of the above studies theoretically analyzed this topic through social combustion theory. In addition, studies ignore the fact that individuals’ communication behavior is also affected by individual differences and the surrounding environment.

Based on the research framework of social combustion theory, this paper crafts a quantitative model for the propagation of public opinion across networks. First, the analogy between public opinion propagation and the combustion phenomenon is established. In this paradigm, individuals within the system are categorically classified into four distinct states, namely, smoldering, combustion, unburned and stable, and the conversion rules between the four types of individuals are given. In addition, the influence of individual differences and the external environment on public opinion dissemination is comprehensively considered, and indicators such as individuals’ self-awareness ability and authority are introduced to reflect individual differences. Concurrently, the influence of surrounding individuals is taken as an external environmental factor, and the whole model is constructed. Subsequently, a large number of simulation experiments are carried out through Monte Carlo simulation. Finally, the rationality and effectiveness of the model built in this paper are verified by real-world case studies.

The remainder of the paper is structured as follows: Section 2 is a literature review; Section 3 describes the research ideas and framework of this paper; Section 4 constructs a network public opinion communication model-DBUS model based on social combustion theory; Section 5 analyzes the factors affecting public opinion communication through simulation experiments; Section 6 verifies the model proposed in this paper with a combination of actual cases; and Section 7 provides a summary of the full text and prospects for future work.

## Section 2: Literature review

Public opinion is the accumulative aggregation of public attitudes and opinions on the occurrence, development, and changes of social events or phenomena in a certain social space. Its communication process can be regarded as the spread and communication of information through relationship networks in the real world and through the Internet. At present, domestic and foreign scholars have accumulated research on the process of online opinion communication, and this section analyzes the literature in terms of the following two aspects: (1) research on the construction of opinion communication models with the help of complex network structural characteristics or infectious disease models; and (2) research on the application of social combustion theory.

### Section 2.1: The construction of opinion communication models

Since the spread of information in social networks is similar to the spread of infectious diseases, most scholars have studied the spread of public opinion with the help of complex network structural characteristics or infectious disease models. The typical literature is as follows. Geng *et al*. [3] integrated the influence of network media and government media on the susceptible-exposed-infectious-removed (SEIR) model. Luo *et al*. [4] conducted a comparative analysis based on two large-scale datasets related to COVID-19 to explore the influence mechanism of sentiment on the forwarding volume. Geng *et al*. [5] simulated the trends of information transmission in the multi-layer network through an improved SEIR model. Zhao *et al*. [6] constructed a SIR model of public opinion propagation based on the novel coronavirus pneumonia model and microblog public health emergency information. Li *et al*. [7] proposed a new public opinion evolution HK-SEIR model that combines opinion fusion HK and epidemic transmission SEIR models. Zhang *et al*. [8] used a modified SIR model to investigate the diffusion effect of information in a coupled social network environment. Xu *et al*. [9] identified a recursive probability function, successfully established a dynamic propagation model based on SIR-I. Yu *et al*. [10] studied the rumor propagation model of heterogeneous networks in a multilingual environment. Zhao *et al*. [11] constructed an emergency information propagation model based on complex network theory. Wang *et al*. [12] proposed a three-party evolutionary game model by identifying stakeholders involved in the process of public opinion communication. Moreno *et al*. [13] derived mean field equations representing the dynamics of the rumor process occurring in complex heterogeneous networks. Zan [14] studied the spread of two kinds of rumors in social networks at different times. In summary, most scholars have built upon existing infectious disease models, incorporating factors such as individual communication willingness and forgetting effects, to analyze the impact of individual differences on public opinion transmission dynamics. Additionally, some researchers have investigated public opinion propagation models in multilingual environments or proposed deterministic mathematical models to explain the spread of public sentiment. However, once public opinion information is received, the public can choose communication or non-communication; however, the aforementioned studies do not separately divide the non-communication population and ignore the influence of netizens on non-communication status.

### Section 2.2: The applications of social combustion theory

The “social combustion theory” first proposed by the Chinese scholar Wenyuan Niu is the result of combining the theory of physics with sociology. The core of the theory is to analogize the combustion phenomenon in the field of natural science with the disorder, imbalance, and turbulence in society, and then to explain and analyze social stability. The harmonious and stable development of society requires effective guidance and intervention in public opinion, considering that the communication of information about public opinion events necessarily goes through the process of incubation, diffusion, and recession, which is in line with the basic model of social combustion theory. Therefore, using social combustion theory as an explanatory framework can aid in the effective analyze of online public opinion communication. Currently, the theory has been widely used, mainly focusing on social stability early warning systems, land dispute risk early warning mechanisms, and the evolution of mass events. Hu *et al*. [15] used social combustion theory to explain the resolution of land disputes and the construction of risk early warning mechanisms in the process of urbanization. Chen *et al*. [16] established a rumor propagation model by comparing users in social networks with burning substances based on the cellular automata model. Wang *et al*. [17] studied the mechanism of online emotional communication in public health emergencies based on social combustion theory. However, the above-mentioned research on public opinion introduced into this theory ignores individual differences due to differences in life experiences, psychology, and regions, as well as whether individuals adopt public opinion communication behavior simultaneously because of the dual effects of the external environment and their ability to resist information.

In summary, scholars have achieved certain results by constructing models to describe the real public opinion communication process. However, a significant portion of these endeavors rely heavily on network structural characteristics and infectious disease models for their insights. In addition, social combustion theory, which is applicable to the study of public opinion communication, is mostly used to study social stability risks, social group events, etc. Only a few scholars have applied this theory in the field of public opinion communication; however, they qualitatively analyze and ignore the fact that public opinion communication is simultaneously affected by individual differences and the impact of the external environment. Therefore, this paper uses social combustion theory to study the process of network public opinion dissemination through building quantitative modeling. Recognizing both individual differences and the external environment, this article introduces indicators such as individual cognitive ability, authority, and conformity to characterize individual characteristics and reflects the role of the surrounding individual influence in the external environment. Finally, through numerical simulation experiments, the key factors affecting the communication of public opinion are identified from the aspects of burning substances, combustion adjuvants, firing temperature, and network structure.

## Section 3: Research ideas and methods

This paper first introduces social combustion theory, which is analogous to the burning phenomenon inherent in network public opinion communication, and finds out that it is reasonable to use social combustion theory to study network public opinion communication. Second, based on the Monte Carlo multi-agent modeling method, this paper uses an Agent to represent a single node in the network and sets the network scale to *N*, i.e., there are *N* netizens in the network. The BA network is taken as the basic network, the framework of social combustion theory is used to analyze the network public opinion communication process, and a simulation experiment is carried out. Finally, the simulation outcomes verify the rationality and effectiveness of the model built in this paper by combining actual cases.

This paper opts to conduct simulations on BA scale-free networks for the following reasons: the BA model has two characteristics: one is the growth characteristic, i.e., new nodes are constantly joining the original network; the second is the merit-based connection feature, i.e., the nodes that have newly joined the network are preferentially connected with those with larger degrees of existing nodes in the network. An online social network is a complex network that conforms to the power law distribution. Its inherent structural properties are similar to those of BA scale-free networks, and the growth characteristics and merit-based connection characteristics of BA scale-free networks are in line with the characteristics of real-life network public opinion propagation [18]. Fan *et al*. [19] studied the network structure and information diffusion of Sina Weibo and found that the topology of Sina Weibo has scale-free characteristics and that the degree distribution obeys a power-law distribution.

**Fig 1:**
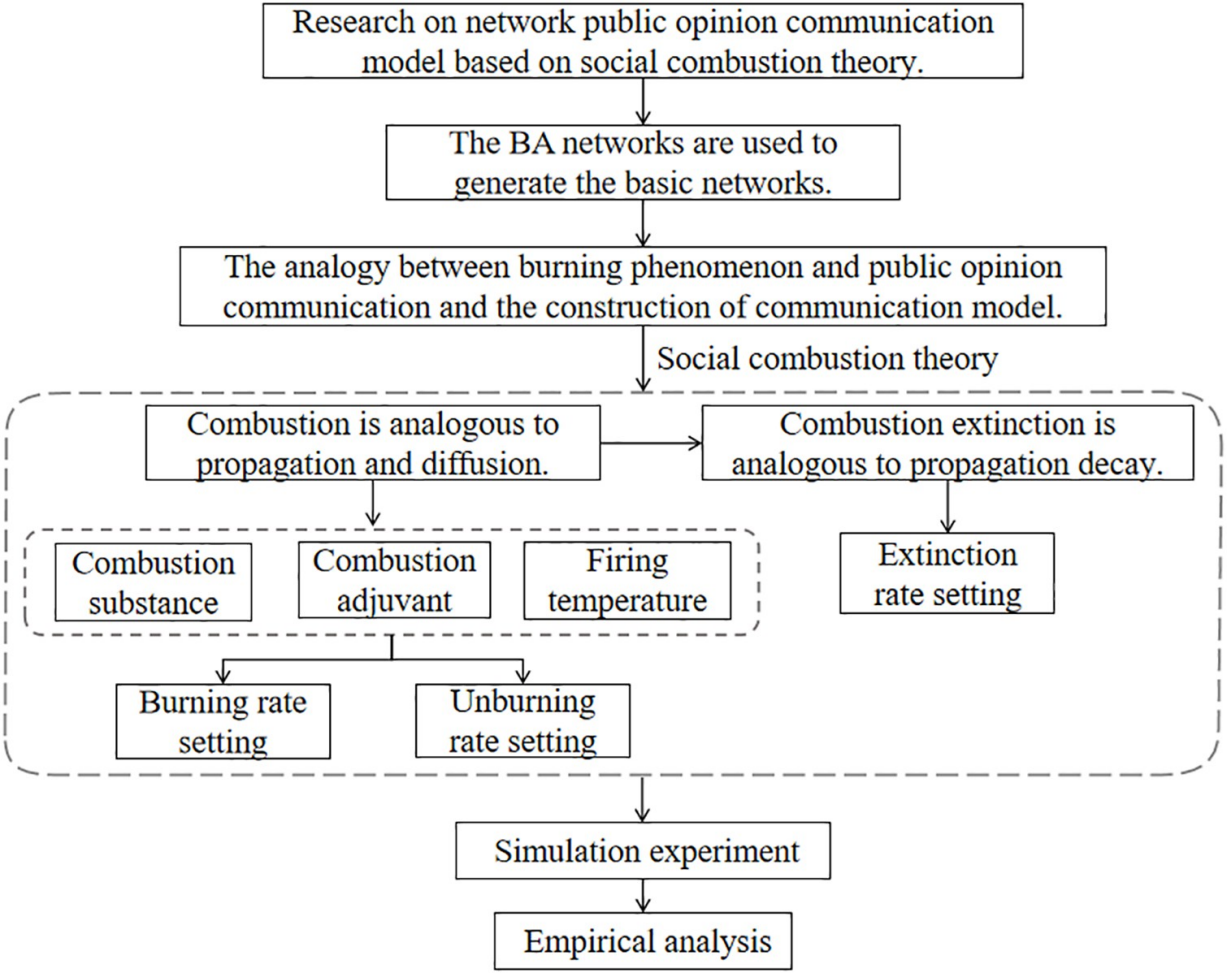
Research framework.

The specific research framework of this paper is shown in Fig 1.

## Seection 4: Construction of the DBUS network public opinion communication model against the background of sudden hot social events

### Section 4.1: The analogy between the burning phenomenon and public opinion communication

Combustion in nature is both a physical and a chemical process, and its production requires three basic elements: combustion substances, combustion adjuvants, and firing temperature. The social combustion theory applies the combustion principle to the analysis of social problems and makes a reasonable analogy between the phenomenon of social disorder and the combustion phenomenon. It is believed that the emergence of social imbalance, disorder, and loss of control also requires the joint action of social “burning substances”, social “combustion adjuvants”, and social “firing temperature”. Inspired by the material combustion process and its principles, according to the laws and actual conditions of public opinion communication, combustion phenomena and public opinion communication exhibit certain similarities through reasonable analogy.

First, from the perspective of the combustion process, combustion must go through three stages: unburning, burning, and extinction. Parallel to life cycle theory, the communication process of network public opinion can also be divided into three stages: incubation, diffusion, and recession. In addition, according to the necessary conditions for combustion, the combustion substances, combustion adjuvant, and firing temperature jointly determine the combustion, and public opinion communication is also affected by various factors. Specifically, in the combustion process, sufficient combustion substances are the basis for the occurrence of combustion phenomena; similarly, netizens are the disseminators and bearers of public opinion information. Additionally, netizens exhibit different behaviors related to public opinion information due to individual differences and different surrounding environments. Only netizens are interested in public opinion information, and public opinion can be disseminated. Second, on the basis of sufficient combustion substances, combustion substances without combustion additives still cannot combust; similarly, the speed of public opinion dissemination and diffusion accelerates when netizens receive relevant public opinion information many times to cause cumulative effects or when the impact of public opinion events themselves is large enough. In addition, in the combustion process, the temperature must surpass a certain ignition threshold to initiate the burning process; similarly, the propagation rate of public opinion information between individuals also has a certain propagation threshold beyond which public opinion information can spread [20]. The mapping between the two influencing factors will be described in more detail later. In summary, public opinion communication and social combustion theory are similar because of the reasonable analogy of the aspects of the combustion process and influencing factors. Therefore, it is reasonable to study network public opinion communication based on social combustion theory, and the mapping process of the two is shown in Table 1.

**Table 1:** Analogy between the spread of network public opinion and social burning.

	Social burning	The spread of network public opinion
State	Unburning	Incubation period
	Burning	Diffusion period
	Extincting	Recession period
Rate of change	The speed of burning	The speed of spreading
Generate conditions	The speed of extinction	The speed of recession
	Multiple factors	Multiple factors
The basis of the phenomenon produced	Combustion substances	Netizens express their views
Promote the emergence of phenomena	Combustion adjuvants	The number of times netizens contact information, the influence of the event itself, etc.
The threshold at which the phenomenon arises	Ignition threshold	Spreading threshold

### Section 4.2: Construction of a communication model based on social combustion theory

#### Section 4.2.1: Group division

**Fig 2:**
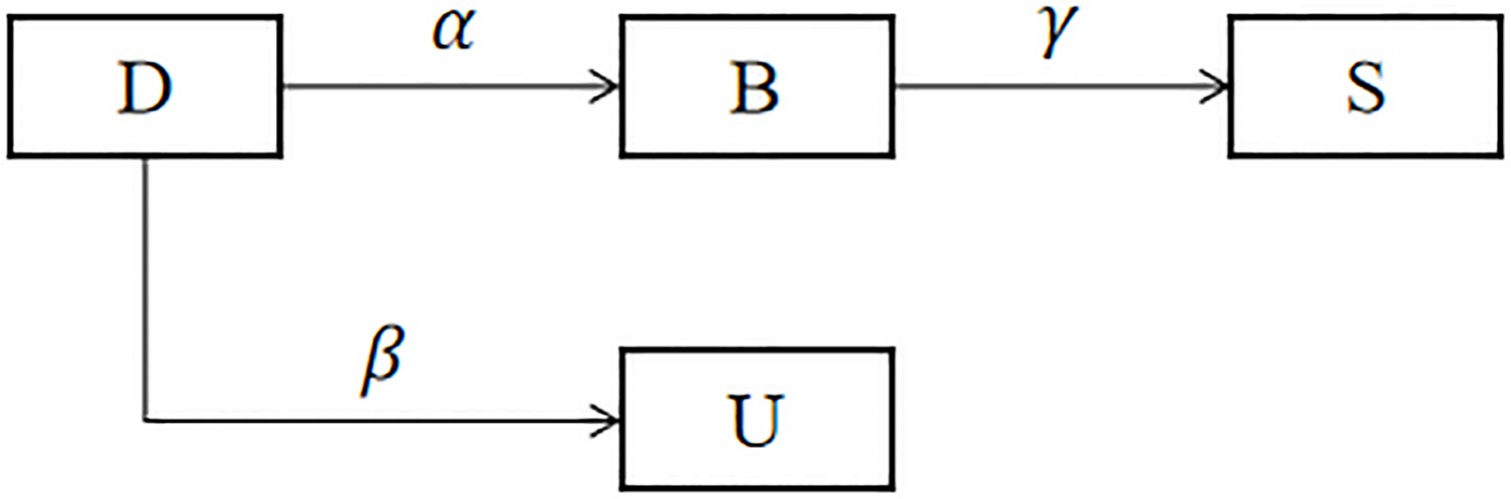
Transition relationships of D, B, U, and S.

In combustion, according to their stability, substances can be divided into divorced substances, burning substances, unburning substances and stable substances [16]. Divorced combustion is a special combustion phenomenon, which refers to the slow burning of a substance without a visible flame, typically producing smoke and an increase in temperature. This phenomenon is distinguished from flaming combustion by the absence of flames. Under the right conditions, such as when there is an ample supply of oxygen, Divorced combustion can rapidly transition to flaming combustion. Combustion refers to a chemical reaction, typically a reaction between a combustible substance and oxygen, which releases heat and light. This process is usually accompanied by the production of flames and can generate smoke, gases, and other by-products. Unburned materials typically refer to substances or fuels that have not yet started to burn. During combustion, combustible materials react with oxygen, generating heat and light to form flames. If certain materials do not engage in this chemical reaction due to reasons such as insufficient temperature, inadequate oxygen supply, or lack of an ignition source, they are considered unburned materials. Stable substances refer to stable compounds formed after combustion. These compounds have sufficient stability and will not further decompose or react under normal conditions. By analogy, users in the social network are divided into divorced (D), burning (B), unburning (U), and stable (S) groups. Divorcee (D) refers to netizens who do not receive public opinion information; Burner (B) refers to netizens who receive public opinion information and spread it immediately; Unburner (U) refers to netizens who receive public opinion information but choose not to spread it; and Stabilizer (S) refers to netizens who have participated in public opinion communication but are no longer interested in it over time. The transition relationships of D, B, U, and S are shown in Fig 2. In Fig 2, *α* represents the burning rate, *β* represents the unburning rate, and *γ* represents the extinction rate.

#### Section 4.2.2: Communication rules

**Fig 3:**
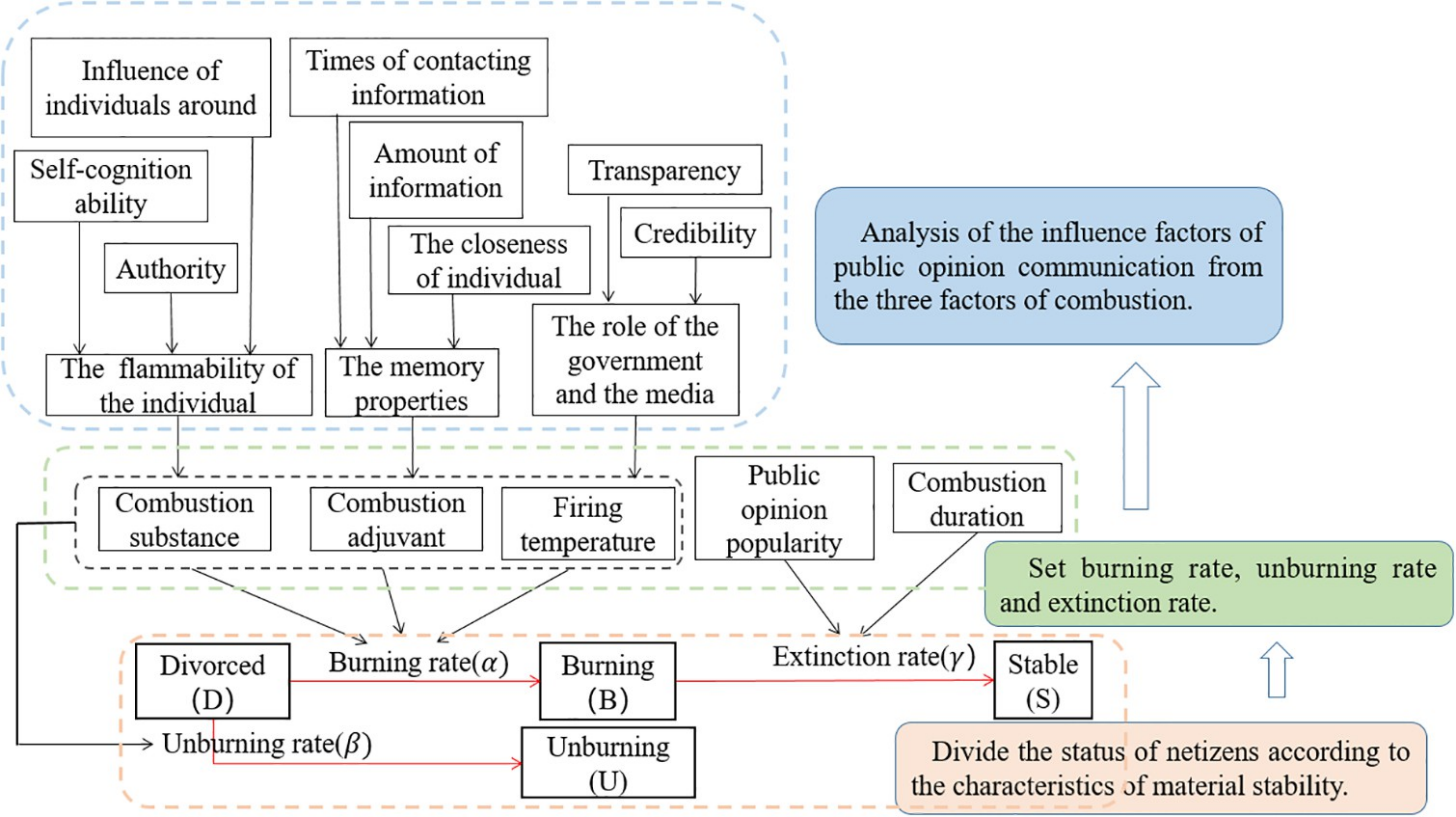
Model construction.

As shown in Fig 3, the communication rules between the four types of communication are as follows:

1. If a divorcee contacts a burner, the divorcee may spread public opinion information at burning rate *α* and turn into a burner due to weak cognitive ability.
2. If a divorcee contacts an unburner, the divorcee may turn into an unburner due to higher authoritative factors and not spread public opinion information with the unburning rate *β*.
3. If a burner contacts a stabilizer, the burner may gradually lose interest in public opinion events over time and turn into a stabilizer with an extinction rate *γ*.

Suppose that in a network composed of *N* individuals, the population densities in the divorced, burning, unburning, and stable states at time *t* are *D*(*t*), *B*(*t*), *U*(*t*), and *S*(*t*), respectively; then, *D*(*t*) + *B*(*t*) + *U*(*t*) + *S*(*t*) = 1. The differential equation of the network public opinion DBUS communication model is as follows:

dDtdt=-αD(t)B(t)-βD(t)U(t)dBtdt=αD(t)B(t)-γB(t)dUtdt=βD(t)U(t)dStdt=γB(t) (1)

### Section 4.3: Function settings

#### Section 4.3.1: Setting burning rate *α*

Section 4.1 states that the public opinion communication process is caused by the combined action of combustion substances, combustion adjuvants and firing temperature; i.e., the combustion rate in the public opinion communication process is related to the three elements of combustion. Generally, more abundant combustion material and stronger excitation and catalytic capabilities of the adjuvant increase the likelihood of combustion. Therefore, according to the literature [21], the combustion rate *α* is defined as follows:

$$\alpha =E_{i}*P(m_{i})*(1-G)$$ (2)

where *E*_*i*_ represents the combustion substance, *P*(*m*_*i*_) represents the combustion adjuvant, and *G* represents the firing temperature.

*(1) Calculation of the combustion substance E*_*i*_. In the process of public opinion communication, individuals both spread and receive public opinion information, which is the basis of public opinion communication; thus, individuals in social networks can be compared to burn substances. In combustion science, substances are flammable and can easily burn or maintain combustion. When users receive public opinion information, considering their individual characteristics and the influence of other users, the possibility of spreading the information varies. Here, the individual characteristics are divided into cognitive ability, authority, and conformity. The reasons are as follows: first, due to differences in education level and social experience, individuals have different cognitive abilities. A stronger cognitive ability enables them to look at public opinion information rationally, and they will not blindly spread this information. Second, opinion leaders in social networks can affect most users. Therefore, individuals with greater authority are more convinced of their views and are less likely to believe and spread public opinion information; i.e., authority inhibits the spread of public opinion. In addition, due to their conformity, when users find that they disagree with most of their neighbors, they will doubt their ideas and gradually tend to agree with others in the network. Therefore, the flammability of individual *i*, *E*_*i*_, is related to the individual’s cognitive ability, authority, and influence of surrounding individuals.

Considering that the impact of public opinion information on individuals is similar to the process of heat absorption by substances [22], the calculation of heat energy is introduced to express an individual’s flammability. *M*_*i*_ represents the effect of the individual’s authority on public opinion communication, i.e., the authority effect. The heat capacity *c*_*i*_ represents the effect of an individual’s cognitive ability on public opinion communication. The temperature *T* represents the influence of surrounding individuals. Based on this, *E*_*i*_ is defined as follows:

$$E_{i}=c_{i}M_{i}\Delta T$$ (3)

Considering that the temperature at the initial moment is 0 for individuals who have not received public opinion information, the calculation formula of flammability *E*_*i*_ can be rewritten as follows:

$$E_{i}=c_{i}M_{i}T_{i}$$ (4)

where *c*_*i*_ represents the heat capacity, *M*_*i*_ represents the mass, and *T*_*i*_ represents the temperature.

Nodes with greater authority tend to attract more attention; these nodes are represented by nodes with a higher degree in the network. Therefore, authority is expressed as the ratio of the degree of individual nodes in the network space to the maximum value of the degree of all nodes in the network. The authority of the largest node in the social network is 1, and the calculation formula is shown in formula (5). In addition, considering the inhibitory effect of authority and cognitive ability on public opinion communication, this paper uses mass *M*_*i*_ and heat capacity *c*_*i*_ to represent the impacts of individual authority and cognitive ability on public opinion communication. Therefore, *M*_*i*_ and *c*_*i*_ are defined as shown in formula (6) and formula (7), respectively.

$$\delta_{i}=\frac{k_{i}}{k_{max}}$$ (5)

$$M_{i}=1-\delta_{i}$$ (6)

$$c_{i}=1-\rho_{i}$$ (7)

where *δ*_*i*_ represents the authority of individual *i*, *k*_*i*_ represents the degree of individual *i*, *k*_*max*_ represents the maximum degree of all nodes in the network, *δ*_*i*_∈(0,1], and *M*_*i*_∈(0,1); *ρ*_*i*_ represents the cognitive ability of the individual, obeying the normal distribution, *ρ*_*i*_∈[0,1]; 0 means that the cognitive level of the individual is the lowest, and 1 means that the cognitive level is the highest.

As a society member, people will be influenced by the surrounding individuals. For individual *i*, if there are more public opinion communicators around, the possibility of changing from an unknown state to a communication state will increase due to the conformity mentality. At this time, the influence of the surrounding individuals will be related to the number of people around the individual who engages in communication behaviors and conformity. Here, *T*_*DBi*_ represents the surrounding individuals’ influence received by an individual who changed from a divorcee to a burner. The calculation formula is shown in formula (8). In the same way, *T*_*DBi*_ represents the surrounding individuals’ influence received by an individual who changed from a divorcee to an unburner. The calculation formula is shown in formula (9).

$$T_{DB_{i}}=con(i)\frac{{NB}_{i}}{k_{i}}$$ (8)

$$T_{DUB_{i}}=con(i)\frac{{NU}_{i}}{k_{i}}$$ (9)

where *con*(*i*) represents the conformity of individual *i*, which obeys a normal distribution, and *con*(*i*)∈(0,1). *NB*_*i*_ represents the number of individuals around whom communication occurs, *NU*_*i*_ represents the number of individuals around whom public opinion information is received but who choose not to communicate, and *k*_*i*_ represents the total number of individuals around. *T*_*DBi*_ represents the influence of surrounding individuals according to formula (8).

*(2) Calculating the combustion adjuvant P(m*_*i*_*)*. In combustion theory, under normal circumstances, if the surface of solid combustibles with the same mass is exposed to more air, the firing energy required for combustion is lower and combustion is more sufficient. The communication of public opinion is affected by the individual memory effect, which is usually related to the number of contacts between individual *i* and relevant public opinion information, the closeness of individual *i* to public opinion events, and the amount of information received by individual *i*. The contact between individual *i* and relevant public opinion information has a cumulative and superimposed effect, especially in the Internet social environment, where individuals can receive public opinion information related to them multiple times at the same time or in a short period. At this time, the contact and the amount of information increase, thereby accelerating the spread of online public opinion. Therefore, the memory properties of individuals are analogous to those of combustion adjuvants. In addition, the communication of online public opinion is affected by the sensitivity of the content. If public opinion events are closely related to the social needs or interests of the public, the content of public opinion is more amenable to communication, and it is easier to trigger public discussion and communication. Based on the above analysis and referring to the literature [23], the individual memory attribute is defined as follows:

$$P(m_{i})=(I_{i}-1)e^{-b_{i}\left(m_{i}-1\right)}+1$$ (10)

where *P*(*m*_*i*_) represents the memory properties of individual *i*, *I*_*i*_ refers to the amount of information received by individual *i* and *I*_*i*_∈[0,1], *b*_*i*_ represents the closeness of individual *i* to public opinion events and *b*_*i*_∈[0,1], and *m*_*i*_ represents the number of contacts between individual *i* and relevant public opinion information.

*(3) Calculating the firing temperature G*. According to combustion theory, combustion material accelerates fermentation under the catalytic action of a combustion adjuvant, after which the temperature further increases, after which the safety threshold is exceeded. At this time, the firing temperature causes the material to burn beyond the safety threshold. The firing temperature generally originates from real events, especially sudden social hot spots. Due to the suddenness of the event, it takes time for the government and the media to judge and identify the event, and extending the recognition and judgment time increases the information ambiguity of the event and raises the degree of public suspicion. At this time, social instability reaches its highest level, breaking through the maximum temperature of social stability and triggering public opinion communication. Therefore, the roles of the government and the media are compared with respect to the firing temperature. In general, if the government and the media have greater credibility and more transparent information, the scope and speed of public opinion communication will be more limited. Based on this, referring to the literature [24], the calculation formula is defined as follows:

$$G=\theta (1-e^{-\varepsilon })$$ (11)

where *θ* represents the transparency of the information released by the government and the media and *θ*∈[0,1] and *ε* represents the credibility of the government and the media and *ε*∈[0,1].

#### Section 4.3.2: Setting unburning rate *β*

In the process of public opinion communication, divorcees may not burn successfully due to a lack of combustion substances, insufficient catalytic ability of combustion adjuvants, failure to reach the combustion threshold, etc., and then choose not to communicate public opinion information. According to the literature [21], the unburning rate *β* is defined here as:

$$\beta =\left(1-E_{i}\right)*\left(1-P\left(m_{i}\right)\right)*G$$ (12)

At this time, the surrounding individual influence used to calculate *E*_*i*_ is *T*_*DUi*_, as shown in formula (9).

#### Section 4.3.3: Setting extinction rate *γ*

In combustion science, when combustion substances and adjuvants undergo oxidation reactions for combustion, the molecules activated by the combustion substances will release a certain amount of energy due to self-decay and eventually lead to self-extinction during combustion. There are similar characteristics in the process of public opinion communication. Due to the rapid development of modern information technology, a large amount of information emerges and is being updated on social platforms such as Weibo every day. With the extension of public opinion communication time, the popularity of public opinion gradually decreases, and individuals in a burning state are no longer interested in content related to public opinion events and gradually change to a stable state. In addition, when most surrounding people are stable, users also lose their interest and motivation to spread public opinion and terminate the spread of public opinion. Therefore, the transition of the combustion state is determined here by the time of public opinion communication and the popularity of public opinion. According to the literature [25], the standard exponential function is used to represent the extension of time, and public opinion popularity is represented by the number of stable state nodes in the surrounding neighboring nodes. The probability *γ* of changing a burner to a stabilizer is as follows:

$$\gamma =1-e^{-(\frac{m(t)}{k_{i}}{+\mu )t}_{1}}$$ (13)

where *t*_1_ represents the time for an individual to become a burner, *m*(*t*) represents the number of stable nodes around individual *i* at time *t*, *k*_*i*_ represents the degree of the node, and *μ* is a fixed constant.

Drawing from the aforementioned insights, this section commences by categorizing netizens into four distinct states with the principles of material stability: divorced, burning, unburning, and stable. The corresponding transition rules for each state are formulated, and the burning rate, unburning rate, and extinction rate functions are subsequently set. This paper states that the burning rate and unburning rate are related to the combustion substances, combustion adjuvants, and firing temperature. The three influencing factors related to public opinion communication are further analyzed, and the entire model is constructed. The idea of model construction is shown in Fig 3. The blue arrow on the right in Fig 3 describes the overall model construction idea, which is divided into three main parts: orange, green and blue. On the left, these three parts are described in more detail, the red arrow section describes the transition rules between the states in detail, and the black arrow represents the mapping relationship between variables and variables and between variables and parameters. For example, a burning substance corresponds to the flammability of an individual, and the flammability of an individual is related to the individual’s self-awareness ability, authority, and influence.

The parameters and variables involved in the process of network public opinion communication are shown in Tables 2 and 3.

**Table 2:** Meaning of parameters.

Sign	Meaning
*ρ*_*i*_	Cognitive ability of individual *i*.
*δ*_*i*_	The authority of the individual *i*.
*con*(*i*)	Conformity of the individual *i*.
*I*_*i*_	Amount of information received by the individual *i*.
*b*_*i*_	The closeness of individual *i* to public opinion events.
*m*_*i*_	Number of contacts between individual *i* and relevant public opinion information.
*θ*	Transparency of information released by the government and the media.
*ε*	The credibility of the government and the media.

**Table 3:** Meaning of variables.

Sign	Meaning
*E*_*i*_	Combustion substance, namely, the flammability of the individual *i*.
*P*(*m*_*i*_)	Combustion adjuvant, namely, the memory properties of the individual *i*.
*G*	Firing temperature, namely, the role of the government and the media.
*D*(*t*)	The proportion of divorcee D in the total number of individuals at time *t*.
*B*(*t*)	The proportion of burner B to the total number of individuals at time *t*.
*U*(*t*)	The proportion of unburner U to the total number of individuals at time *t*.
*S*(*t*)	The proportion of the stabilizer S to the total number of individuals at time *t*.
*α*	Burning rate
*β*	Unburning rate
*γ*	Extinction rate

**Fig 4:**
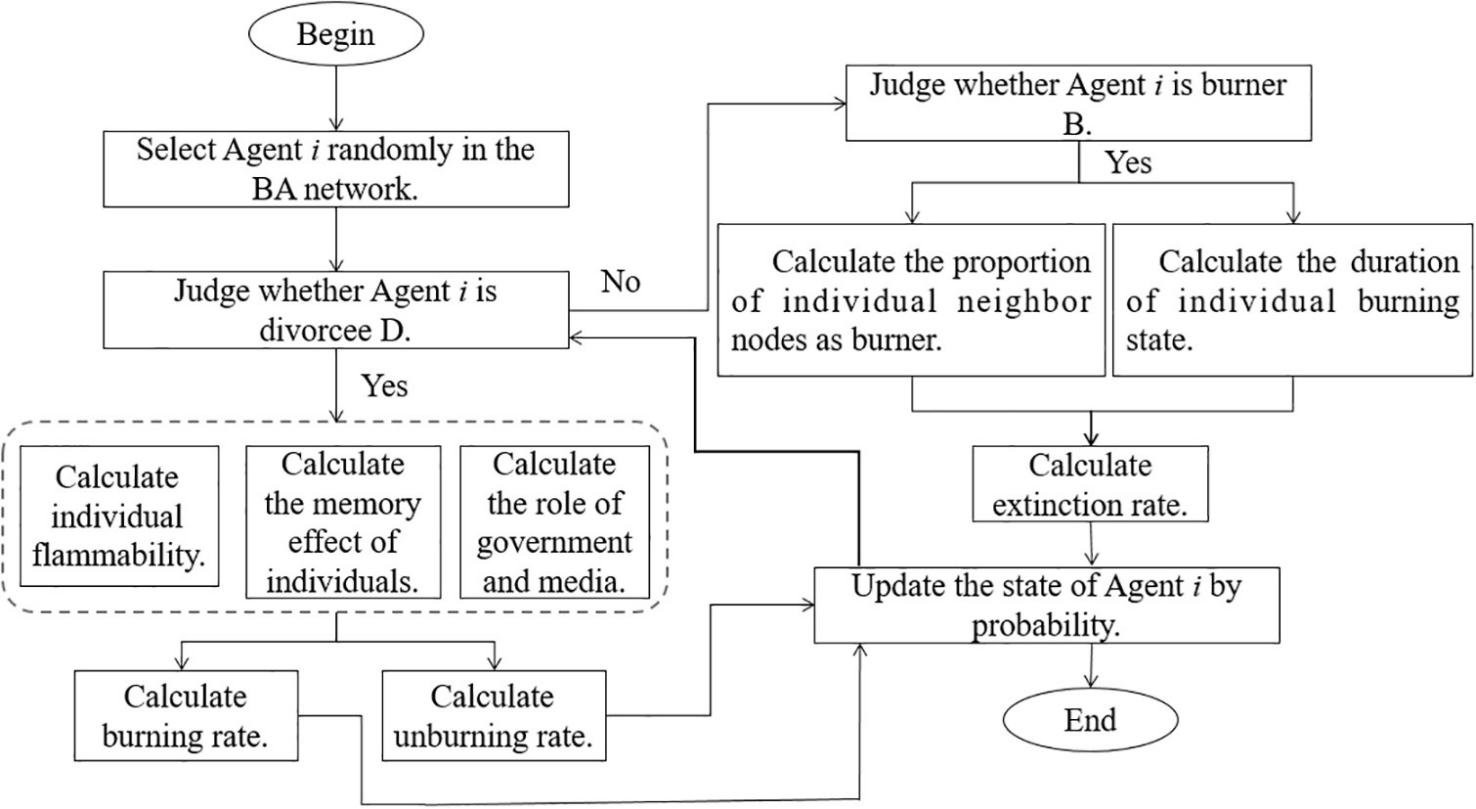
Flowchart of the network public opinion DBUS communication model.

A flow chart of the network public opinion DBUS communication model constructed here is shown in Fig 4.

## Section 5: Simulation experiment

In this section, MATLAB software is used to simulate and analyze the aforementioned constructed model. In the model, public opinion communication is affected by three factors: combustion substances, adjuvants, and firing temperature. Therefore, this section first explores the impacts of individuals’ cognitive ability, authority, conformity, amount of information received by the individual, relationship between public opinion events and netizens, credibility of the media and the government, and transparency of the information released on the process of public opinion communication. Then, to determine the key factors, a combination and comparative analysis of the above factors is carried out; finally, the influence of different network topologies on public opinion communication is analyzed. The initial network of the simulation experiment is a BA scale-free network [26], and the node size *N* is set to 1000. Considering comprehensive visualization, the number of burners at the initial moment is set to 1, and the rest are divorcees, *ρ*~*N*(0.5,0.2), *I*~*N*(0.5,0.2), *b*~*N*(0.5,0.2), *θ =* 0.6, *ε =* 0.8, *m =* 5, *μ =* 0.01, and the evolution time is set to *T* = 100. To avoid contingency of the experimental results, each group of experiments is measured by the average value of 100 simulation results.

### Section 5.1: Impact of substances on public opinion communication

#### Section 5.1.1: Impact of cognitive ability *ρ*_*i*_ on public opinion communication

**Fig 5:**
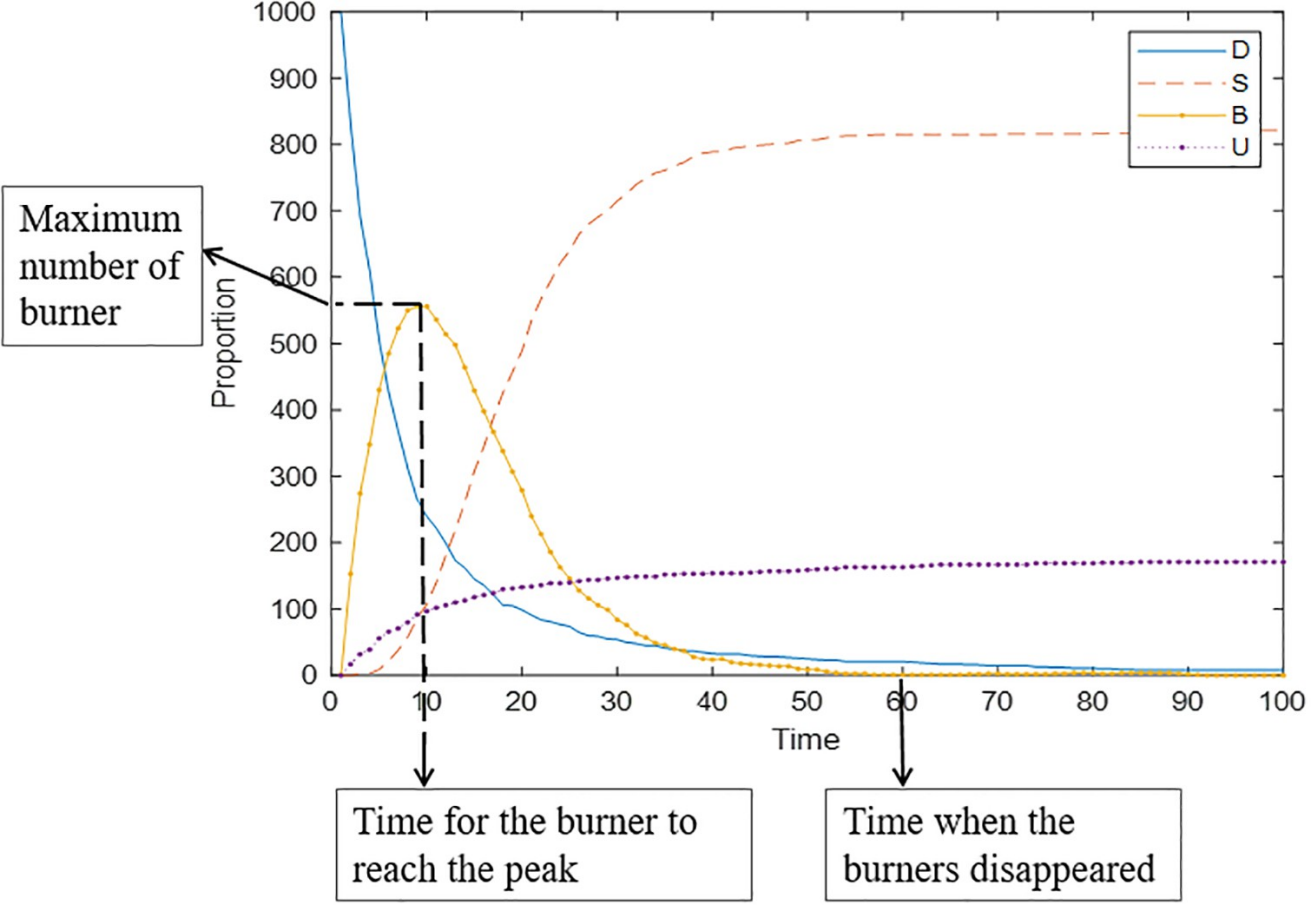
Changes in the maximum number of burners, the time needed for the burner to reach the peak, and the time when the burners disappeared over time.

Cognitive ability can measure an individual’s ability to distinguish public opinion information. Generally, the stronger an individual’s self-cognition ability is, the more rationally he/she can treat public opinion information, and the less likely he/she is to adopt public opinion communication behavior. Based on this, a simulation analysis of the public opinion communication process under different cognitive abilities is carried out here. Setting *ρ*_*i*_ to obey the normal distribution of *N*~(0.2,0.2), *N*~(0.4, 0.2), *N*~(0.6,0.2), and *N*~(0.8,0.2) and the time for the burner to reach the peak, the maximum number of burners and the time when the burners disappeared are used as three indicators reflecting the communication speed, communication range, and dissipation speed of public opinion, respectively. The results are shown in Fig 5.

**Fig 6:**
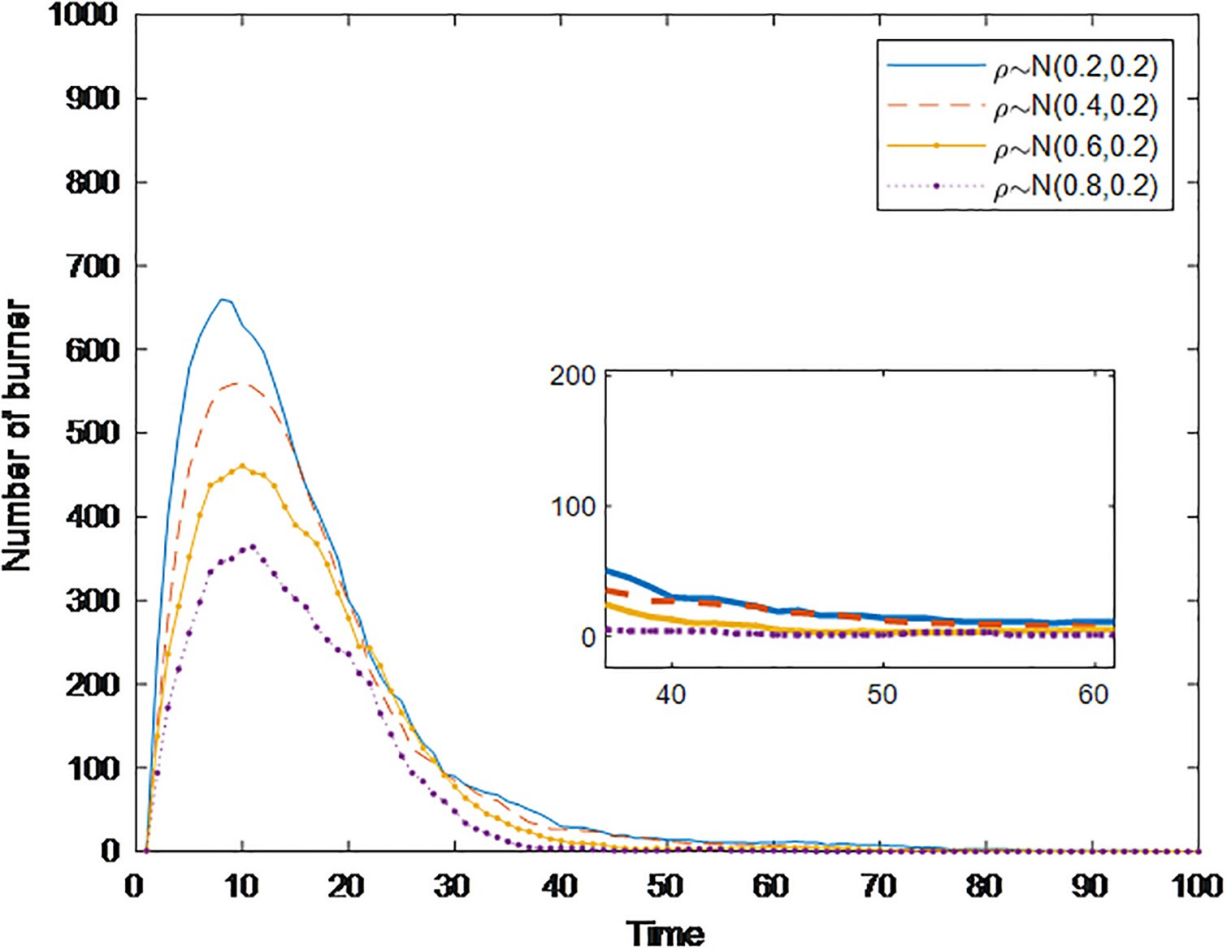
The number of burners with different cognitive abilities over time.

Fig 6 shows the time-varying graph of the number of burners under different cognitive abilities. The small part of the graph in Fig 6 is a partially enlarged view of the moment when public opinion is about to dissipate, to more intuitively compare the effect of cognitive ability on the speed of public opinion dissipation. Fig 6 shows that with the improvement of cognitive ability, the burners’ time to reach the peak is extended, i.e., the communication speed of public opinion slows; at the same time, the peak value that can be achieved is greatly reduced, i.e., the communication range is significantly narrowed. In addition, the time needed for the number of burners to decrease to 0 is also gradually shortened, i.e., the speed of public opinion dissipation is accelerated. In summary, individuals’ cognitive ability can reduce the speed and narrow scope of public opinion communication and accelerate its dissipation. This conclusion is the same as in the study of Li [27]. Individuals with strong cognitive abilities need time to think rationally and have a strong ability to distinguish information. Although some of these individuals also participated in the communication, information about the event is continuously revealed over time, so people could stop the dissemination in a timely manner and become stable.

#### Section 5.1.2: Impact of authority *δ*_*i*_ on public opinion communication

**Fig 7:**
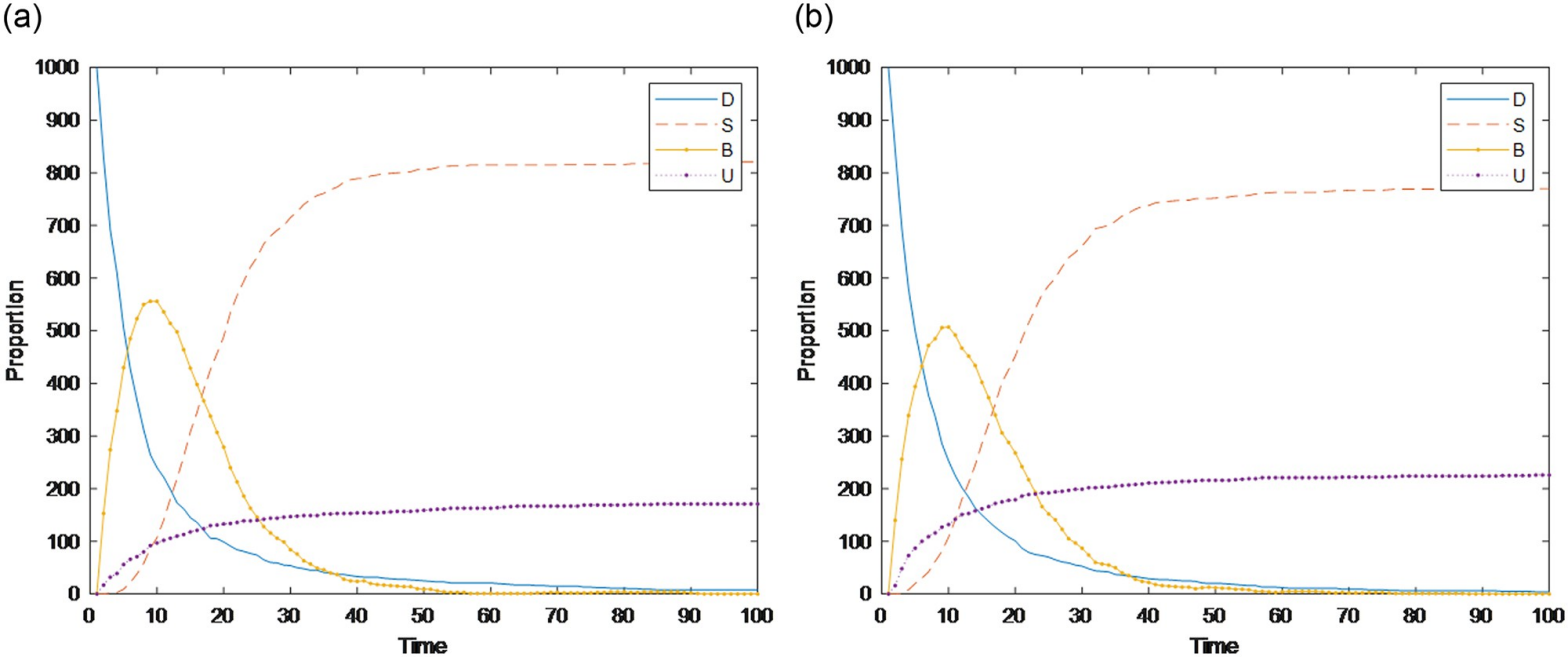
Changes in individuals while considering individual authority. (a) Without considering individual authority, (b) Considering individual authority.

Generally, individuals with greater authority are less likely to participate in discussions of public opinion events. Here, two sets of experiments are used to compare the impact of individual authority characteristics on public opinion communication. The simulation results are shown in Fig 7. Fig 7(a) shows that without considering the authority of the audience, the time for the burner to reach the peak is 10, the peak reaches 515, and the time node for it to decrease to 0 is approximately 50. Compared with Fig 7(b), when considering the authoritative characteristics of the audience, i.e., *M*_*i*_ is not 1, under the same initial environment, the time for the burner to reach the peak and the time when the burners disappear remain unchanged, but the maximum scale reached is narrowed, indicating that after the introduction of authority characteristics in the public opinion communication model, it has a greater impact on individual nodes with higher authority. This conclusion is the same as in the study of Li [27]. In social networks, there is a phenomenon of opinion leaders, which means that an authoritative user has high social influence, and their social account often attracts a large number of followers. Their words and actions can influence the majority of users, such as Weibo celebrities, which are uneasily influenced by external opinions and will pay attention to their own words and deeds. It is not easy to believe and forward public opinion information; thus, the range of audience members exposed to information through celebrities decreases, and there are fewer nodes in the burning state; i.e., the authoritative characteristics of the audience narrow the spread of public opinion.

#### Section 5.1.3: Impact of conformity *con(i)* on public opinion communication

**Fig 8:**
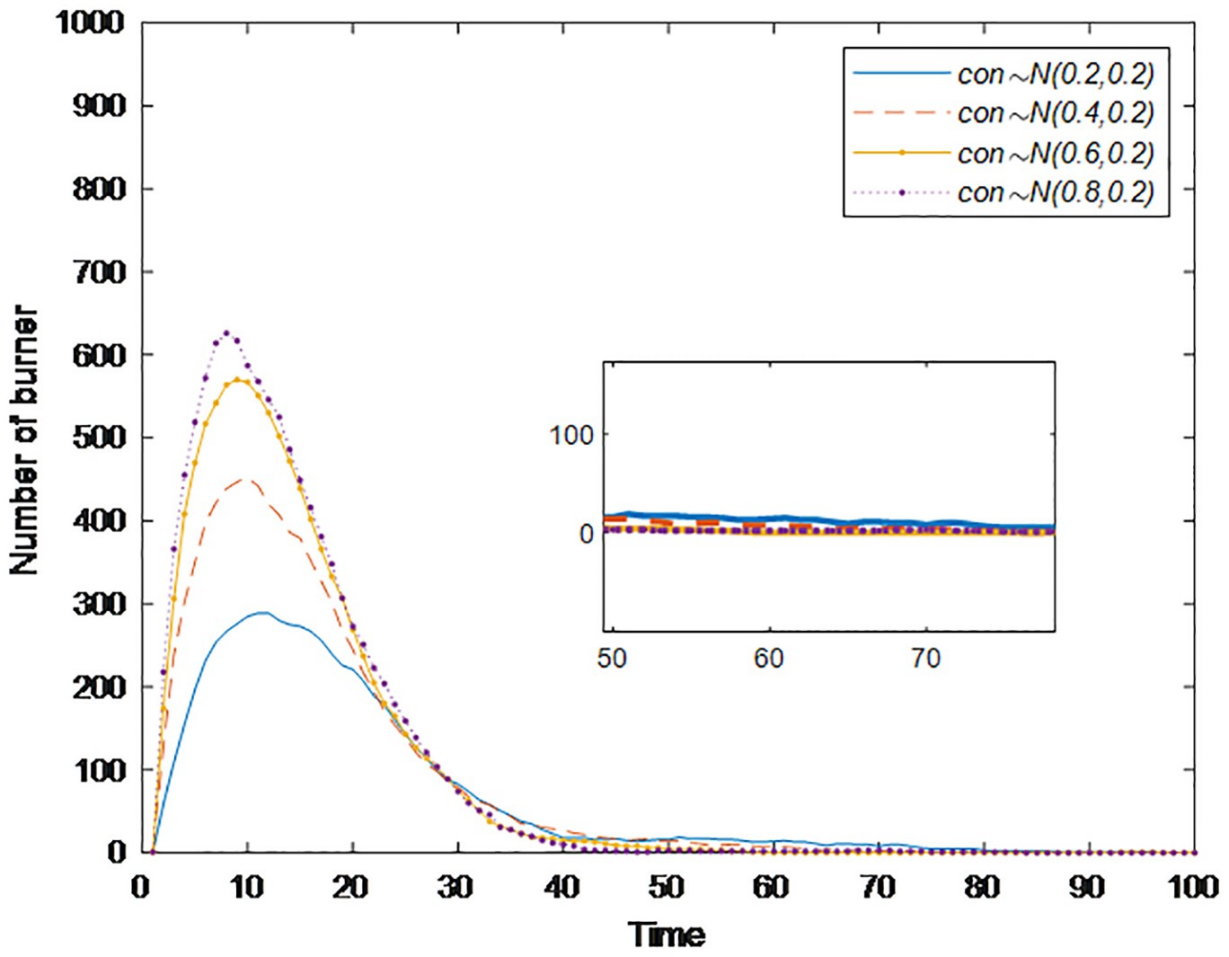
The number of burners under different conformity conditions over time.

People living in a group society are influenced by their surrounding people, and conformity can measure the influence of individuals nearby. Generally, people with a greater degree of conformity are more affected by their surroundings. Based on these findings, a simulation analysis of the public opinion communication process considering the degree of conformity of different individuals is carried out to explore its impact on the communication speed, communication range, and dissipation speed of public opinion. Fig 8 shows the changes in burners under different conformity degrees, in which the conformity degree *con*(*i*) obeys the normal distribution of *N*~(0.2,0.2), *N*~(0.4,0.2), *N*~(0.6,0.2), and *N*~(0.8,0.2). Fig 8 shows that with increasing conformity degree, the time needed for the burner to reach the peak is gradually shortened, but the peak value significantly increases from 289 to 621; i.e., the range of public opinion communication is significantly expanded. Moreover, the time when the number of burners is reduced to 0 is gradually shortened, i.e., the speed of public opinion dissipation accelerates. The reason may be that the people surrounding them lose interest in this public opinion event. Individuals with a higher degree of conformity no longer participate in the discussion of this event, and then quickly transform into stabilizers without spreading public opinion information. This conclusion is the same as in the study of Wang [21]. In social networks, when communicating public opinion information, individuals may doubt and change their views, judgments, and behaviors due to their characteristics and the influence of surrounding groups. The conformity mentality greatly expands the scope of public opinion communication and accelerates the speed of its spread, but at the same time, the speed of its dissipation accelerates.

### Section 5.2: Impact of combustion adjuvants on public opinion communication

#### Section 5.2.1: Impact of the amount of information received by individual *I*_*i*_ on public opinion communication

**Fig 9:**
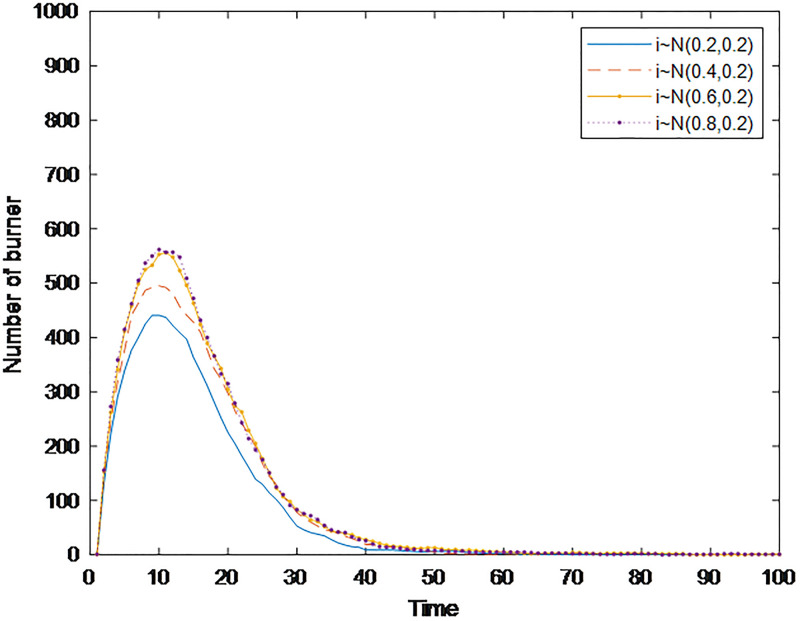
The number of burners under different amounts of information over time.

In the era of the mobile Internet, the low-cost information exchange of online platforms enables information to spread faster and more widely than ever before. Based on these findings, a simulation analysis of the public opinion communication process involving different types of information received by individuals is carried out. The results are shown in Fig 9. With the increase in the amount of information received by the individual, the time for the burner to reach the peak and the time when the burners disappear change slightly, indicating that the amount of information does not impact the communication and dissipation speed of public opinion. Furthermore, when *I*_*i*_ increases to a certain value, the increase is extremely small, indicating that the amount of information can improve the communication range of public opinion; however, when *I*_*i*_ increases to a certain value, the effect is weakened. As public opinion events unfold, rumors and other divergent opinions continue to emerge, sparking the interest of netizens. Netizens actively engage in discussions about the events, leading to an increase in the volume of information and promoting the dissemination of public opinion. However, after some time, as the truth is gradually revealed, some individuals regain their rationality and no longer participate in the discussions about the events.

#### Section 5.2.2: Impact of the correlation degree *bi* between public opinion events and netizens on public opinion dissemination

**Fig 10:**
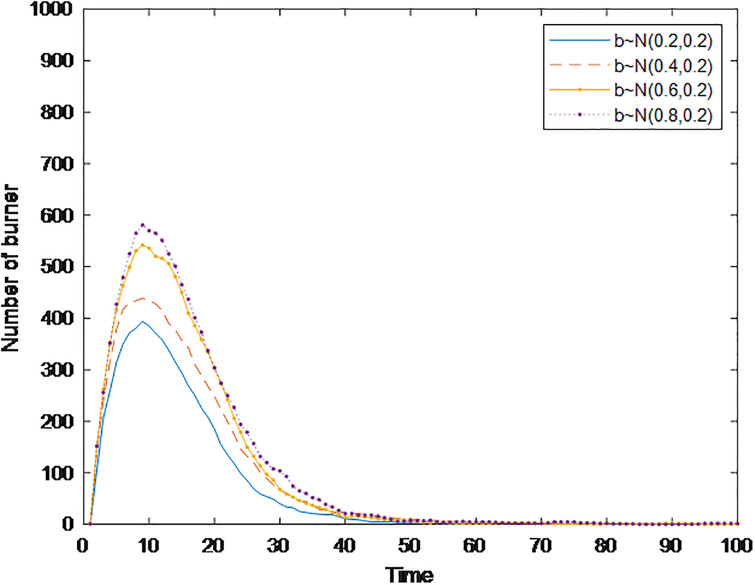
The number of burners under different closeness conditions over time.

Generally, when a public opinion event involves the interests of netizens, it is easier to attract their attention and stimulate individuals to participate in the discussion of the event and promote the communication of public opinion. Based on this, a simulation analysis of the network public opinion communication process under different closeness degrees is carried out. Fig 10 shows the number of burners with time when *b*_*i*_ obeys *N*~(0.2,0.2), *N*~(0.4,0.2), *N*~(0.6,0.2), and *N*~(0.8,0.2). As shown in Fig 10, with increasing closeness degree, the time for the burner to reach the peak and the time when the burners disappear change slightly, but the maximum scale reached by burners expands, indicating that closeness does not affect the communication and dissipation speed of public opinion. This conclusion differs from Jiang et al. [28]. However, in real life, public opinion events are often closely related to people’s lives, such as food safety incidents, once they occur, they will inevitably trigger intense discussions among the public.

#### Section 5.2.3: Impact of the number of contacts between individuals and public opinion information on public opinion communication

**Fig 11:**
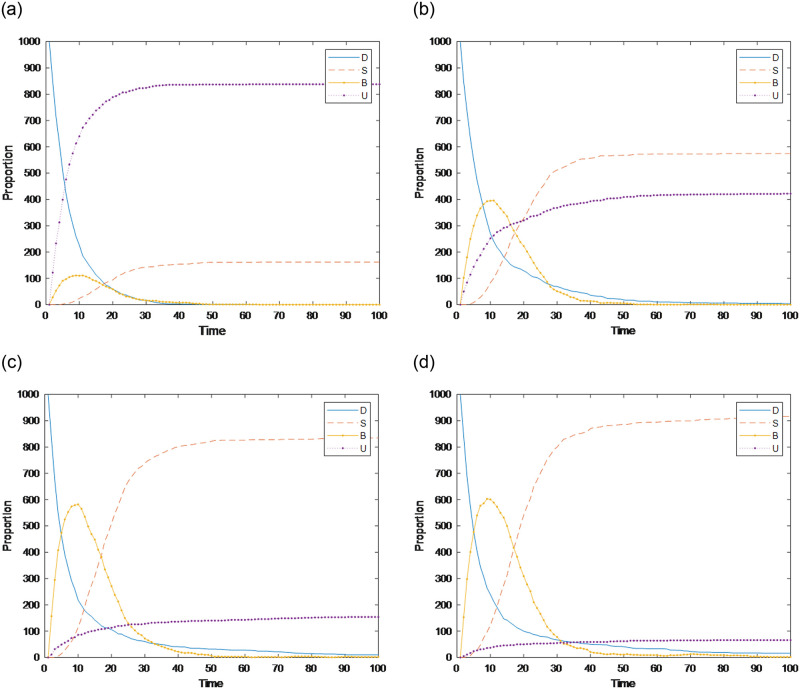
The number of burners under different contact numbers over time. (a) *m* = 1, (b) *m* = 3, (c) *m* = 6, d) *m* = 9.

In the current social networking environment, it is easy for netizens to receive the same public opinion information from different channels many times in a short period. This short-term memory strengthens multiple ways, prompting people to communicate. Based on this, a simulation analysis of the number of contacts involved in public opinion communication is carried out, setting *m* = 1, *m* = 3, *m* = 6, and *m* = 9. The results are shown in Fig 11. It can be seen from Fig 11(a)–11(d) that with increasing *m*, the time for the burner to reach the peak does not change significantly, but the time when the burners disappear gradually increases, indicating that the number of contacts between individual *i* and relevant public opinion information does not significantly impact the communication speed of public opinion but delays the dissipation of public opinion. In addition, a comparison of Fig 11(a)–11(c) reveals that when the number of contacts increases from 1 to 6, the maximum scale at which the burner can significantly increase from 112 to 588. From Fig 11(c) and 11(d), when *m* increases from 6 to 9, its peak value increases only slightly, indicating that the number of contacts can expand the range of public opinion communication; however, when *m* reaches a certain value, the effect weakens. This conclusion is the same as in the study of Wang [21]. The dissemination of information is influenced by the audience’s memory effect, meaning that exposure to information has a cumulative effect. Especially in the online social environment, individuals in social networks can simultaneously or repeatedly receive public opinion information related to events in a short period of time, which reinforces the audience’s memory, thereby increasing the probability of information dissemination.

### Section 5.3: Impact of firing temperature on public opinion communication

#### Section 5.3.1: Impact of credibility *ε* on public opinion communication

**Fig 12:**
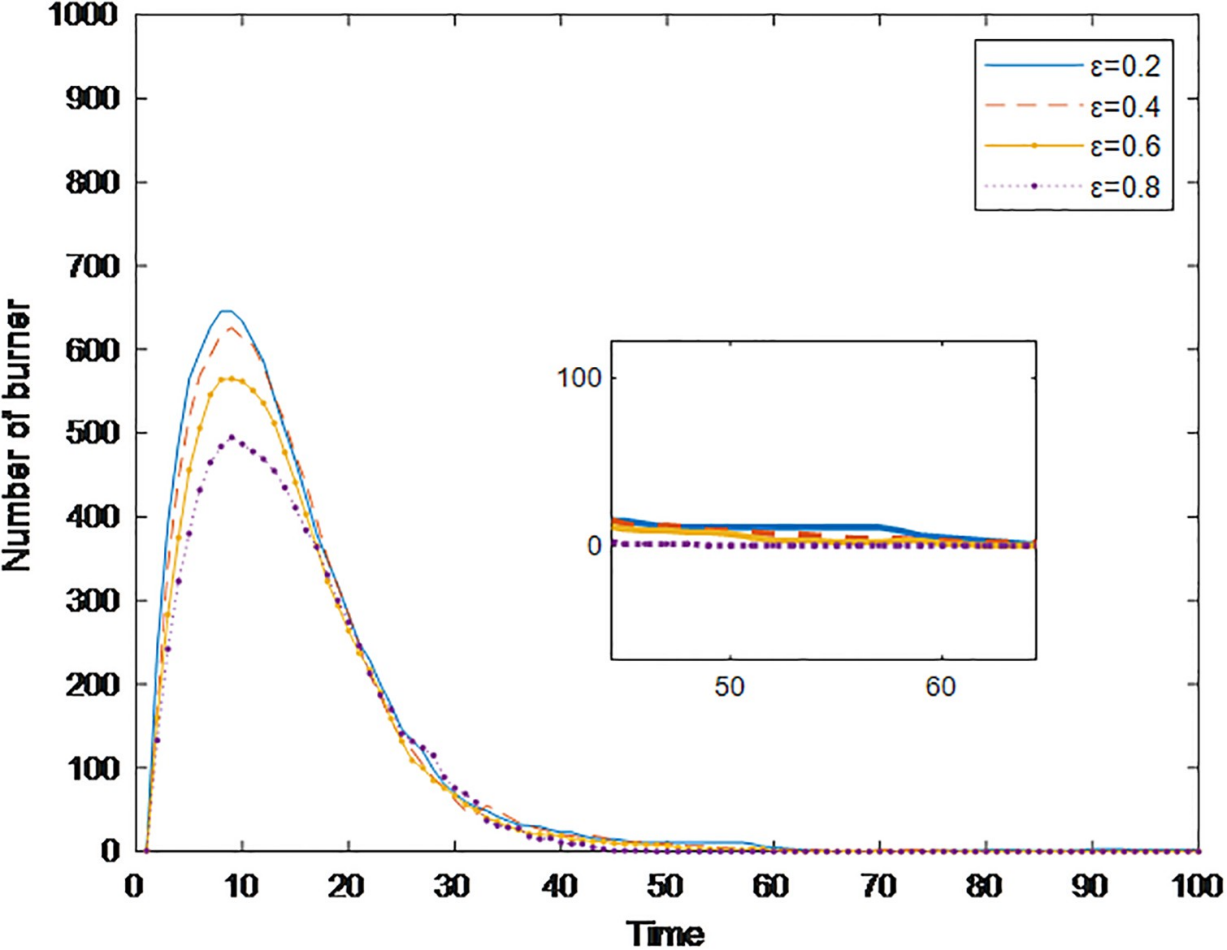
The number of burners under different credibilities over time.

To effectively prevent the fermentation of public opinion, the government usually releases information about an incident at an appropriate time when the credibility of the government and the media will affect its ability to suppress public opinion communication. Generally, greater government and media credibility helps netizens accept their views more easily. Low credibility promotes netizens to have a rebellious mentality and spread information. Therefore, this paper explores the impact of the government and media on public opinion communication in social networks with different levels of credibility. The simulation results in Fig 12 show the change in the number of burners with time when *ε* = 0.2, *ε* = 0.4, *ε* = 0.6, and *ε* = 0.8. As shown in Fig 10, with increasing *ε*, the time for the burner to reach the peak changes slightly, but its peak value decreases, and the time when burners disappear decreases, indicating that the credibility of the government and the media insignificantly impacts public opinion communication; however, this change reduces the range of public opinion communication and reduces the amount of time needed for public opinion dissipation. This conclusion is the same as in the study of Zhao *et al*. [11]. The reason may be that when the credibility of the government and the media is high, netizens will be more willing to accept their views. At this time, the government clearly guides public opinion.

#### Section 5.3.2: Impact of transparency of information *θ* on public opinion communication

**Fig 13:**
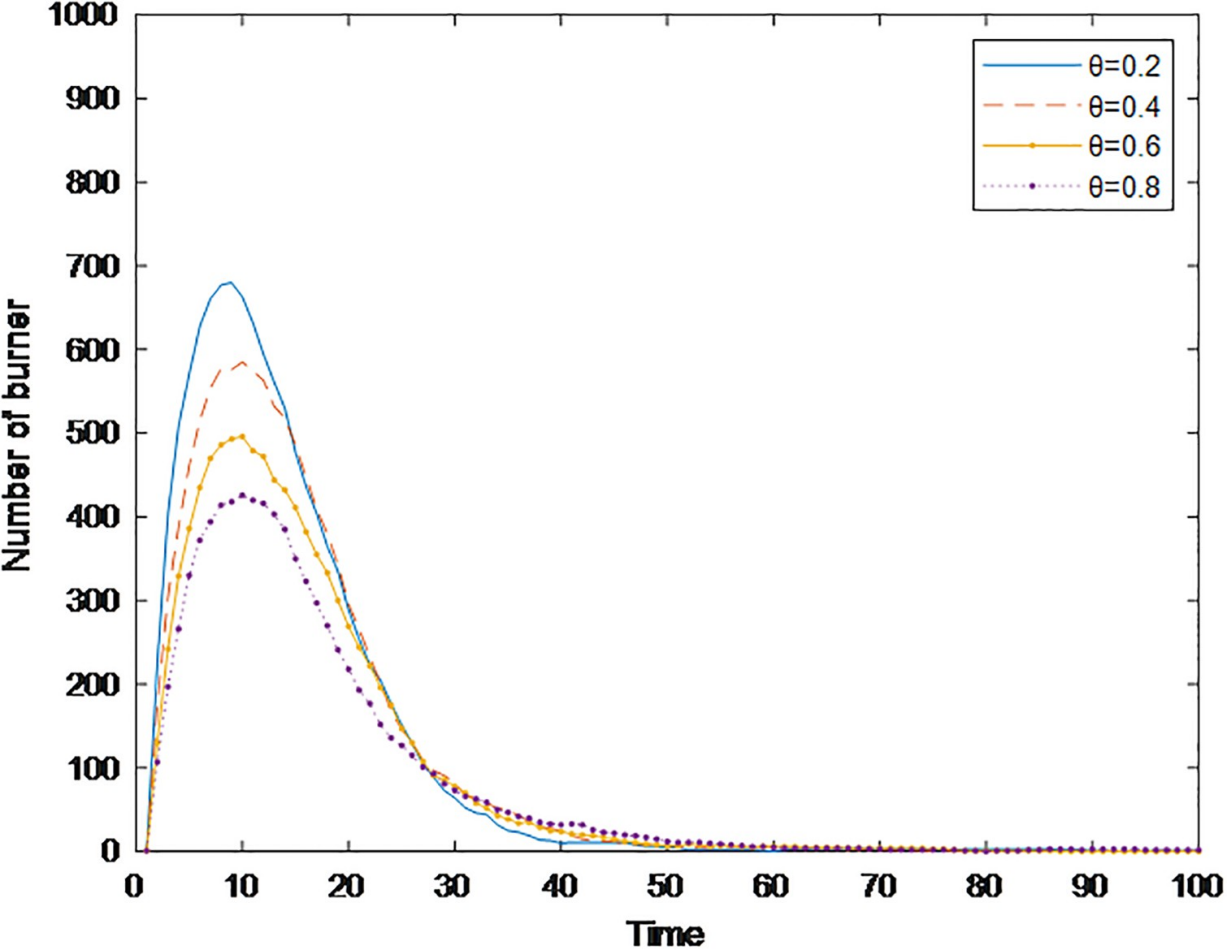
The number of burners under different transparencies over time.

People are often eager to obtain the truth. At this time, if the government and the media do not release or withhold information in time, it will arouse netizens’ suspicion, stimulate their curiosity, and prompt them to actively participate in discussions, thereby promoting public opinion communication. Based on these finding, a simulation analysis of the public opinion communication process under different levels of transparency of information released by governments and media is carried out to explore its impact on public opinion communication. The results are shown in Fig 13. With increasing transparency, the peak value reached by the number of burners decrease significantly from 689 to less than 415, but the time to reach the peak and the time when burners disappeared change slightly. This shows that the transparency of information released by the government and the media has no impact on the communication or dissipation speed of public opinion, but can narrow the communication range of public opinion. This conclusion is the same as in the study of Li et al. [2]. The higher the transparency of information released by the government, the netizens can recognize the nature of information, and reduce the generation of alienated remarks such as rumors to a certain extent, so as to reduce the interest of netizens in this event and reduce the spread of public opinion.

### Section 5.4: Impact of combined factors on public opinion communication

#### Section 5.4.1: Combination analysis of factors affecting the speed of public opinion communication

**Fig 14:**
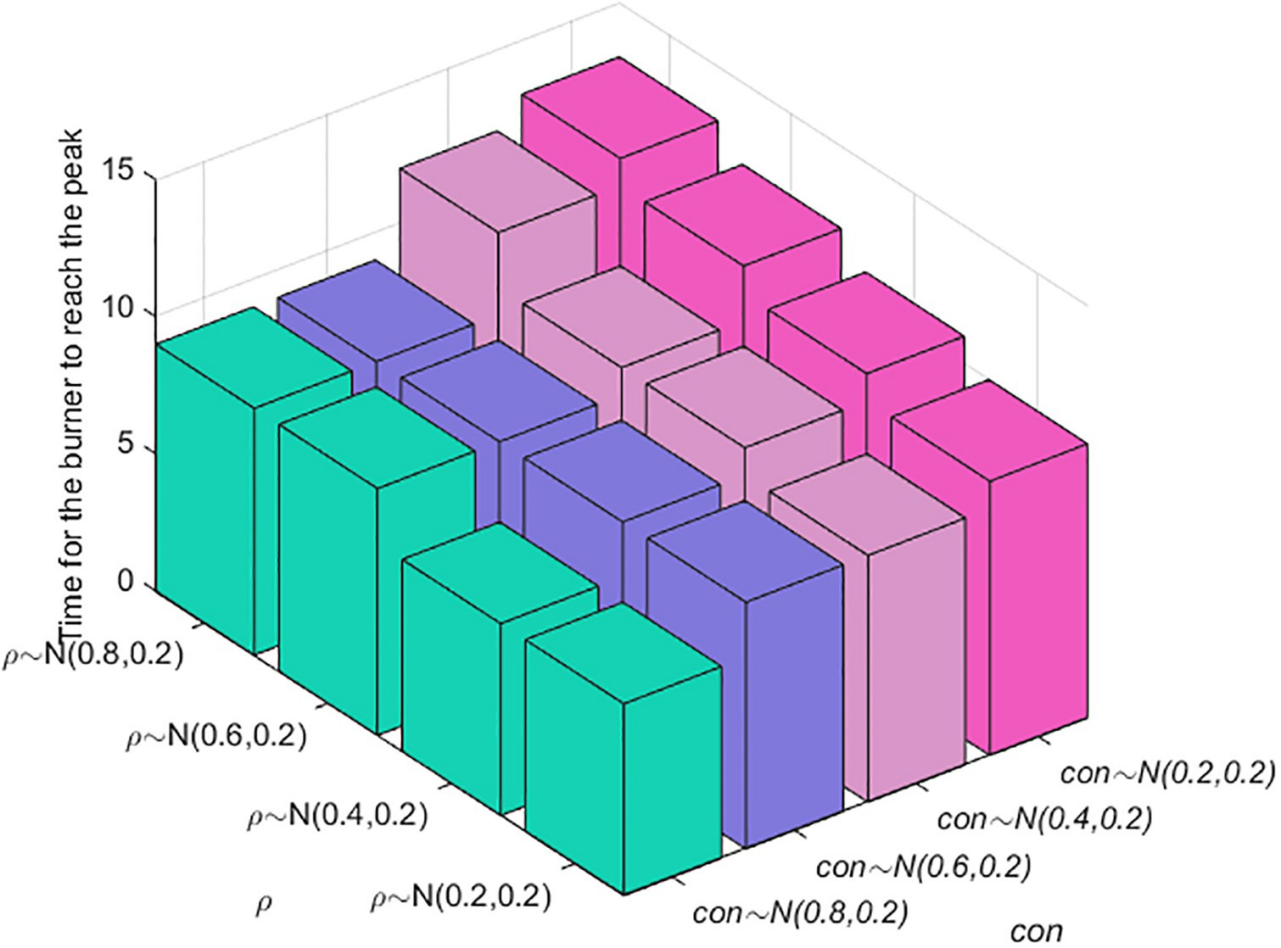
Combination analysis of factors affecting the speed of public opinion communication.

The above simulation analysis reveals that the factors affecting the speed of public opinion communication include two main factors: an individual’s cognitive ability and conformity. Here, a combined analysis is carried out to determine the key factors. The results are shown in Fig 14. With the increase in individual conformity and the decrease in cognitive ability, the time needed for burners to reach the peak increases; i.e., the speed of public opinion communication increases. When the conformity degree is *con*(*i*)~*N*(0.6,0.2), as cognitive ability improves, the time for the burner to reach the peak does not change; however, as the conformity degree increases, the time to reach the peak changes. Compared with cognitive ability, an individual’s degree of conformity has a greater impact on the speed of public opinion communication.

#### Section 5.4.2: Combination analysis of factors affecting the speed of public opinion communication

**Fig 15:**
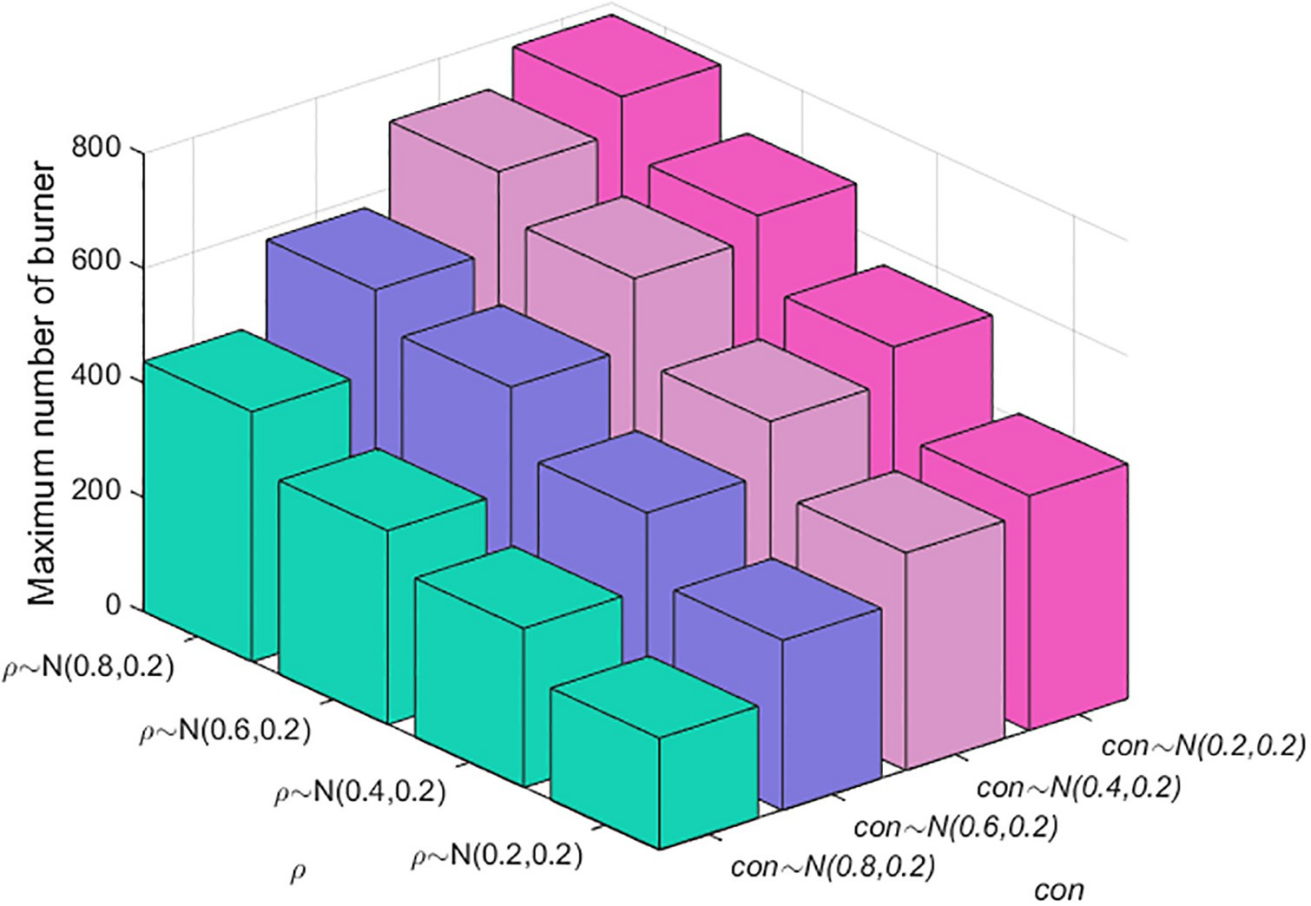
Relationships among individual cognitive ability, conformity and public opinion communication.

**Fig 16:**
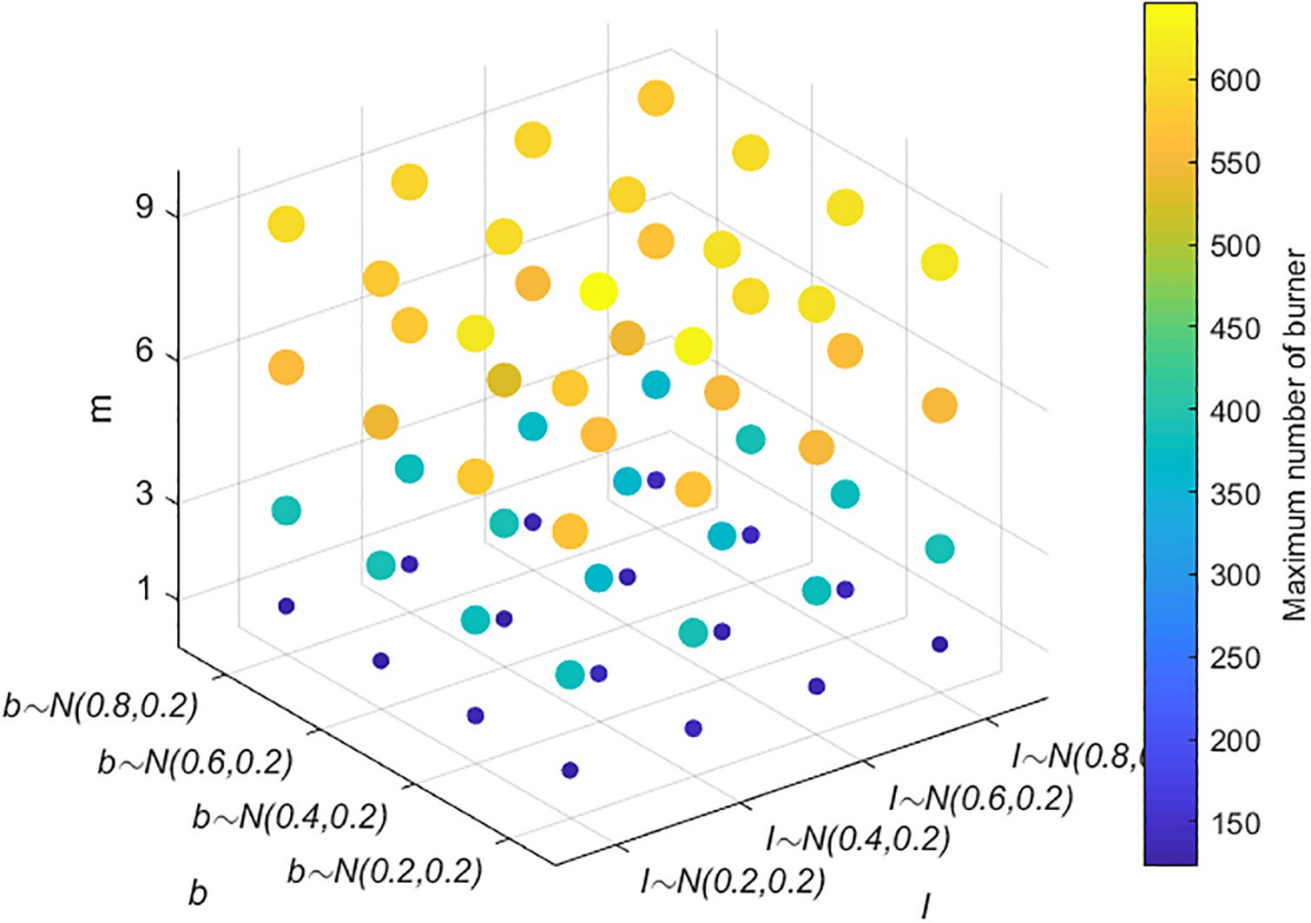
Relationships among the amount of information, closeness, the number of times information is contacted and public opinion communication.

**Fig 17:**
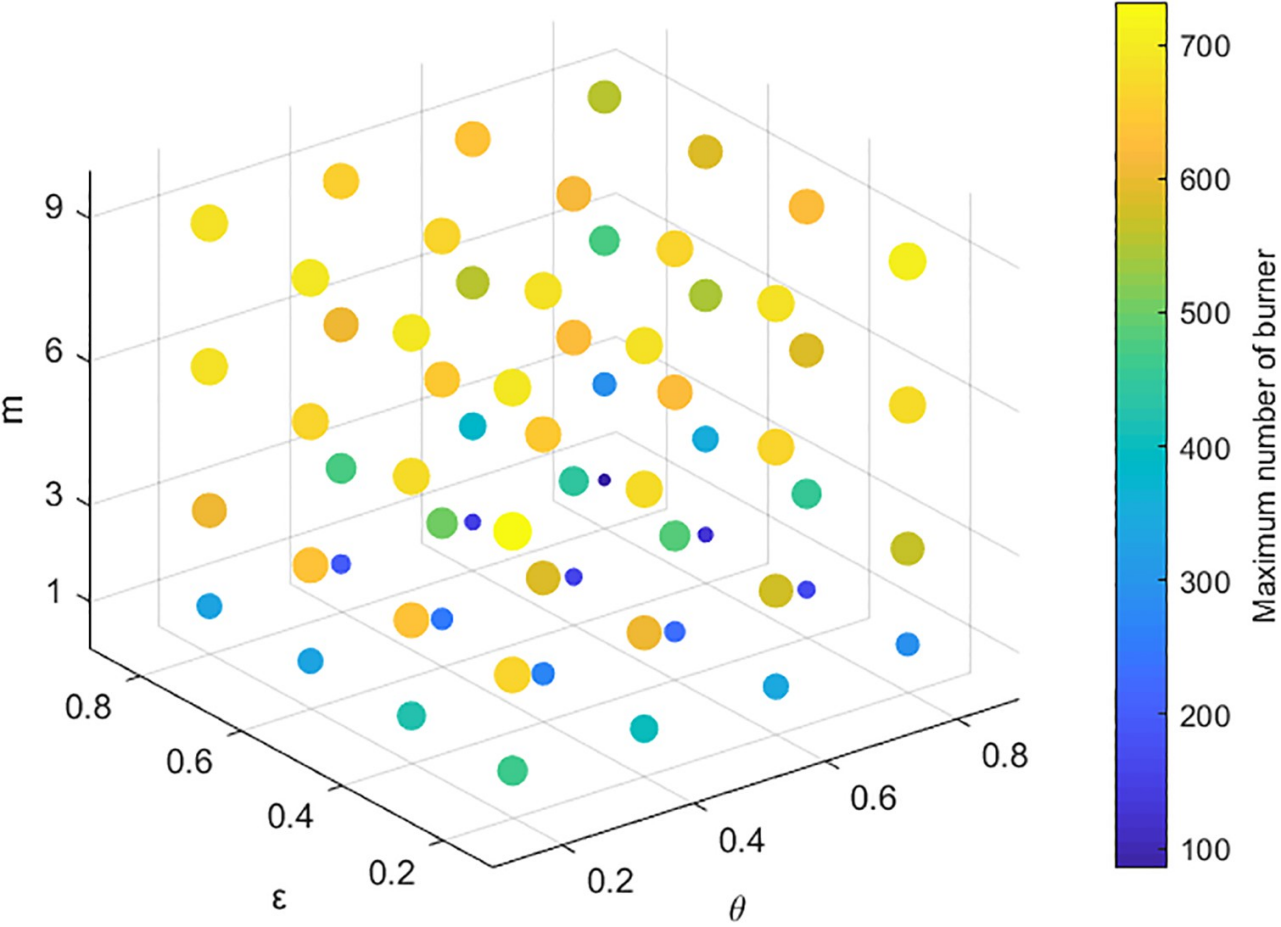
Relationships among credibility, transparency, the number of times information is contacted and public opinion communication.

**Fig 18:**
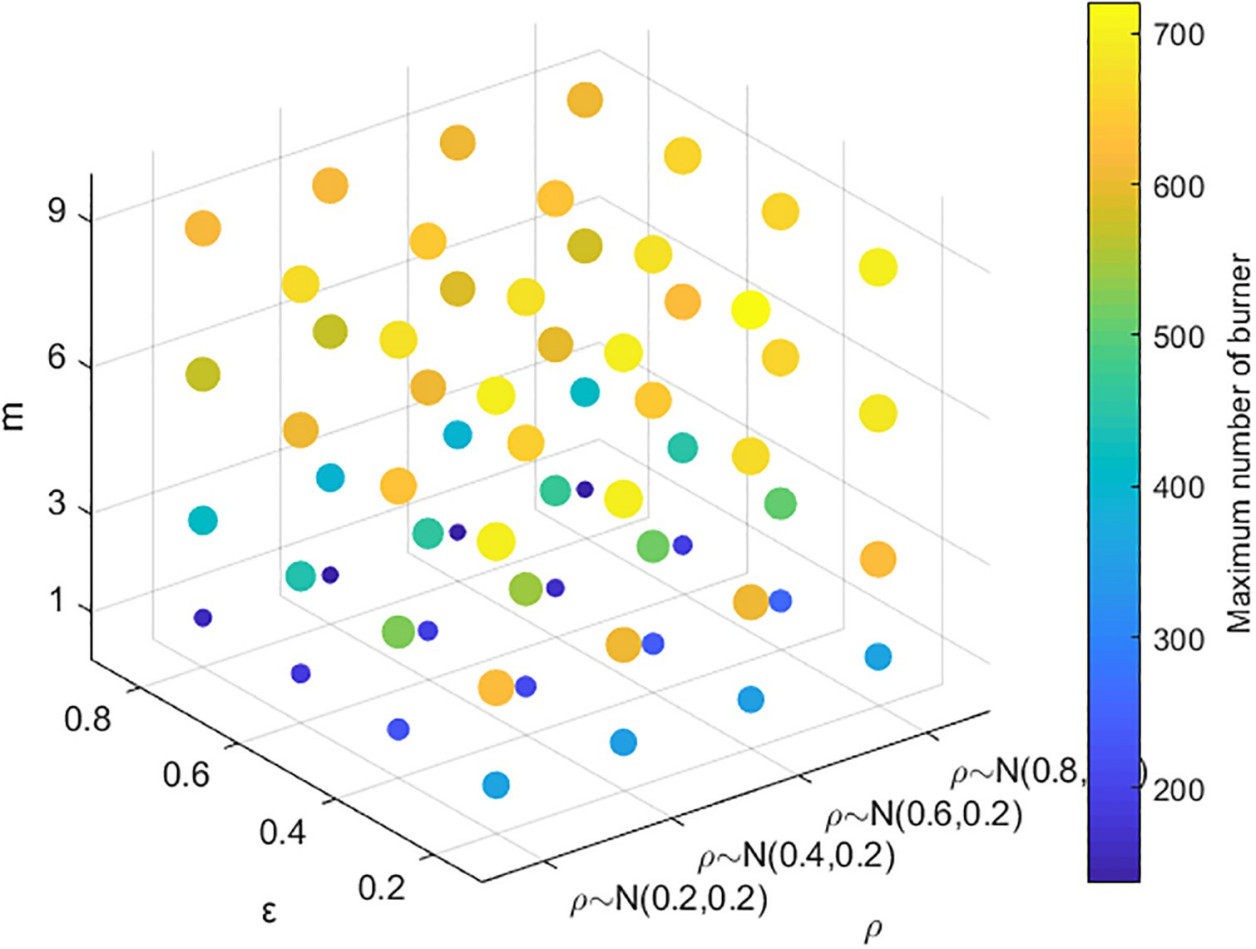
Relationships among cognitive ability, credibility, the number of times information is contacted and public opinion communication.

The above simulation analysis demonstrates that many factors affect public opinion communication. In the real evolution of public opinion, due to the urgency of network public opinion management and control, it is usually necessary to focus on key links to reduce the cost of public opinion prevention and control. Therefore, identifying the most critical factors among the many factors affecting public opinion communication is more in line with practical needs. Here, the key factors are identified by combining and analyzing the factors affecting the range of public opinion communication. The results are shown in Figs 15–18. In Figs 16–18, the larger scatter and lighter color indicate that the burner reaches a larger peak, i.e., public opinion spreads wider.

Figs 15–18 show the relationships among the combinations of individual cognitive ability, conformity, information amount, closeness, the number of times information is contacted, transparency, and credibility of information released by the government and the media, and public opinion communication. Fig 15 shows that with improved individual conformity and weakened cognitive ability, the peaks reached by burners increase, but the increases in the two are not much different. This shows that the influences of conformity and cognitive ability on the range of public opinion communication are almost the same. Fig 16 shows that with an increase in the amount of information, correlation, and the number of times of information is contacted, the maximum number of burners increases; however, when the amount of information and closeness increase, the color and size of the scatter change less and even remain unchanged when the number of times information is contacted are less than 3. Under different contact information durations, the color and size of the scatter change obviously, which shows that, compared with the amount of information and the closeness, the communication scale of public opinion is more affected by the number of contact information points. As shown in Fig 17, as the credibility of the government and the media increase and as the amount of information released increase, the range of public opinion communication narrows sharply. When the number of contacts increases to a certain threshold, the range of public opinion communication expands, indicating that compared with the number of contacts, the credibility of the government and the transparency of information have greater impacts on the range of public opinion communication. Fig 18 shows that with decreasing credibility and increasing number of times information is contacted, the range of public opinion communication expands sharply. However, with the continuous increase in individual cognitive ability, the peak value reached by the burner decreases only slightly. The figure shows that the size of the scatter plot and the color change slightly. Therefore, compared with individuals’ cognitive ability, their credibility and the number of times they contact information have greater impacts on the range of public opinion communication. In summary, the credibility of the government and transparency of information are the most critical factors affecting the range of public opinion communication, followed by the number of contacts, cognitive ability, and conformity.

#### Section 5.4.3: Combination analysis of factors affecting the speed of public opinion dissipation

**Fig 19:**
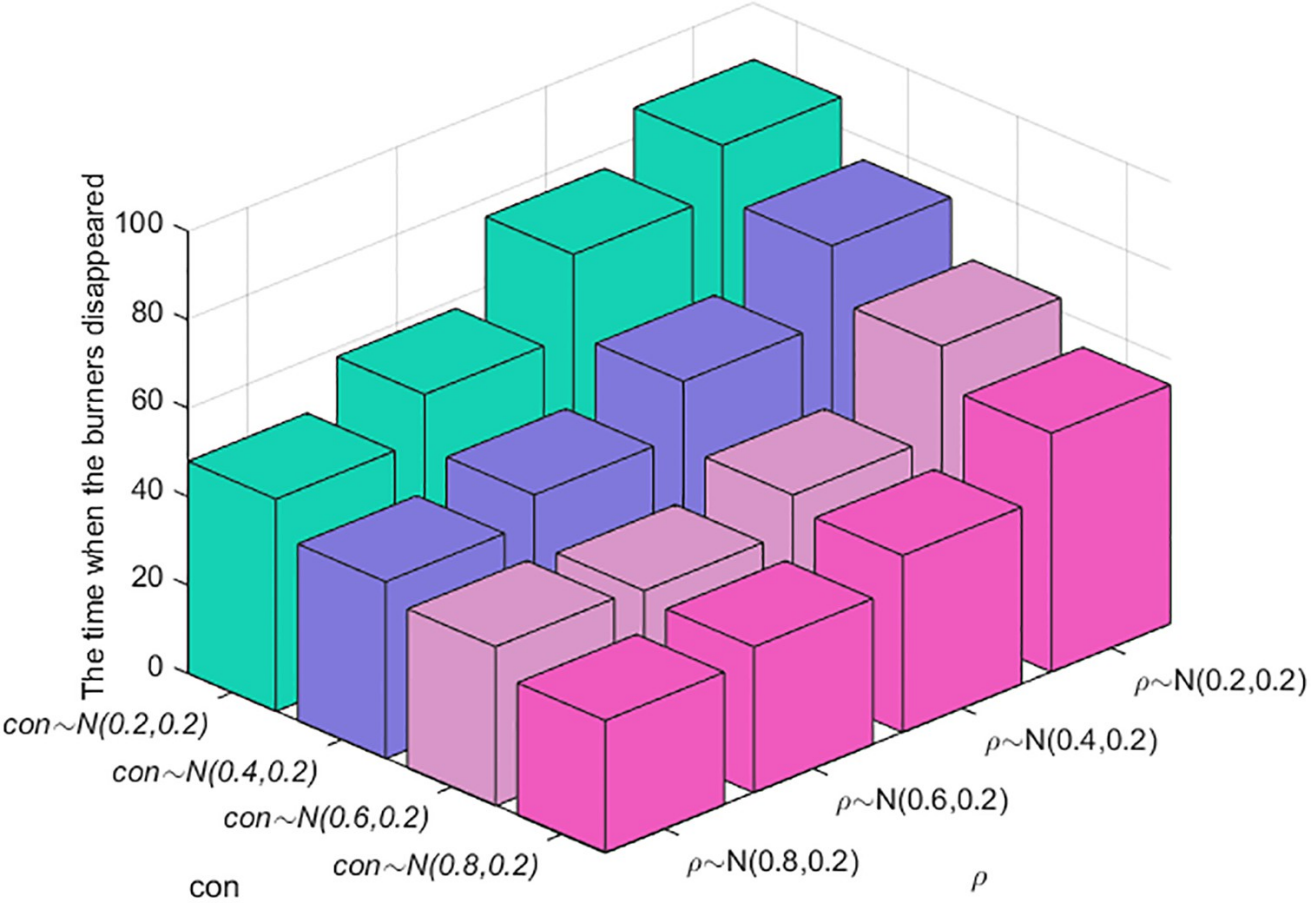
Relationships among individual cognitive ability, conformity and public opinion dissipation.

**Fig 20:**
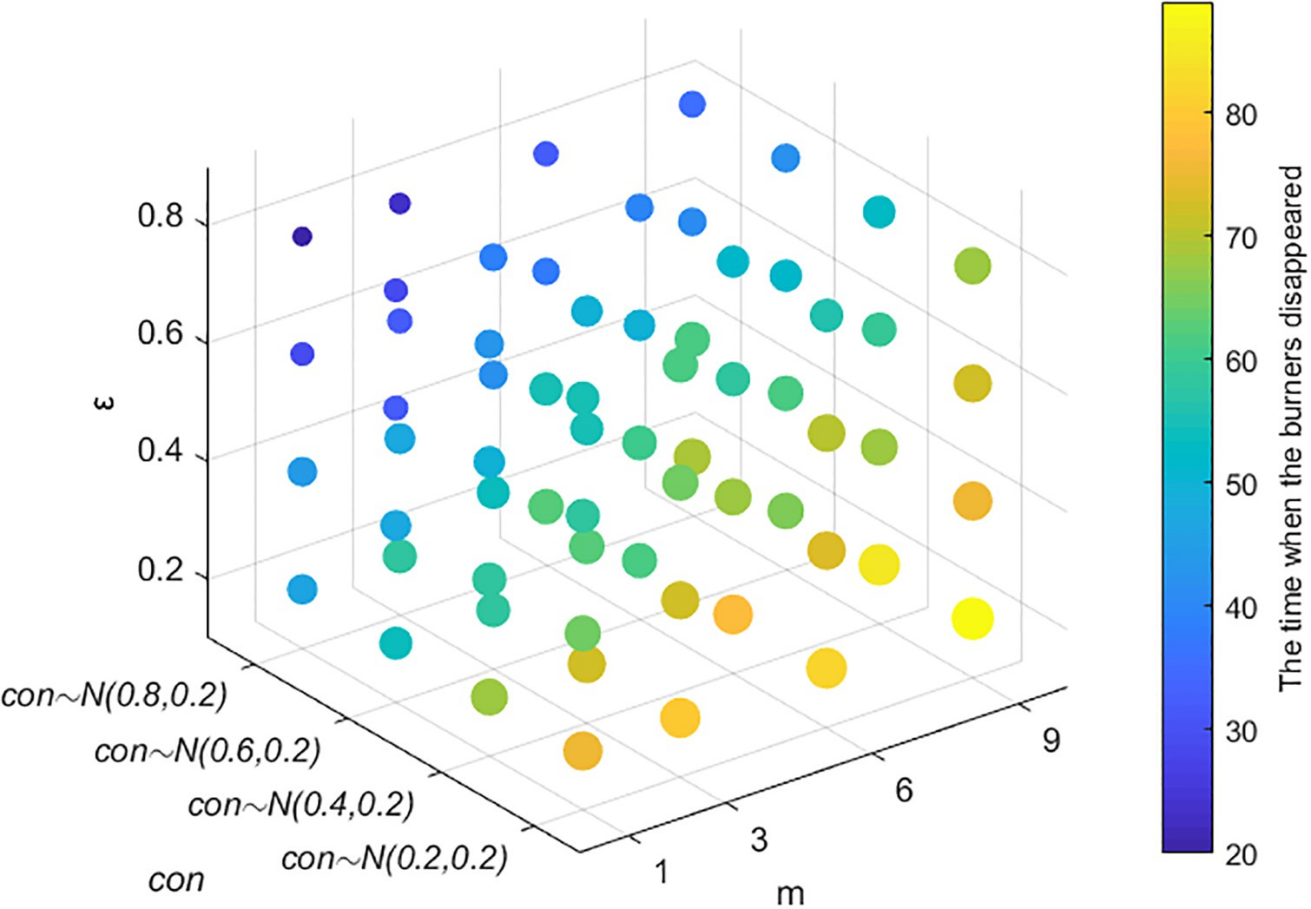
Relationships among conformity, the number of pieces of receiving information, credibility and public opinion dissipation.

The above simulation analysis reveals that the factors affecting the dissipation of public opinion include four main factors: an individual’s cognitive ability, conformity, the number of times information is contacted, and the credibility of the government and the media. To analyze the data in a comprehensive manner, a combined analysis is carried out. The results are shown in Figs 19 and 20. A smaller scatter and darker color in Fig 20 indicate that burners are less likely to disappear and that the dissipation of public opinion is faster.

As shown in Fig 19, as individuals’ cognitive ability and conformity improve, the amount of time when the burner disappears decreases; i.e., public opinion dissipation accelerates, and the changes are consistent, indicating that the impacts of cognitive ability and conformity on public opinion dissipation are almost the same. As shown in Fig 20, greater conformity and credibility and fewer instances of receiving information indicate that the dissipation of public opinion is faster. When the conformity of individuals and the credibility of the government and the media change, the time required for public opinion to completely dissipate does not substantially differ. When the credibility *ε* obeys *N*~(0.6,0.2) or *N*~(0.8,0.2), and the conformity degree obeys *N*~(0.6,0.2), as the number of times information is received increases, the dissipation speed of public opinion does not change significantly, indicating that compared with the amount of contact information, an individual’s conformity and the credibility of the government and the media have greater impacts on the dissipation speed of public opinion.

### Section 5.5: Impact of network structure on public opinion communication

The spread of online public opinion is carried out through social networks. As a complex network, the structure of social networks has an important impact on the dissemination of public opinion information [29]. In this paper, the BA scale-free network, WS small-world network [30] and all-connected network [31] are selected for comparative analysis. The topological structure parameters of each network are shown in Table 4.

**Table 4:** Topological structure parameters of each network.

The name of the network	Average path length	Cluster coefficient	Average degree
BA scale-free network	4.05146	0.02429	3.968
WS small-world network	6.98848	0.27013	4
All-connected network	1	1	999

**Fig 21:**
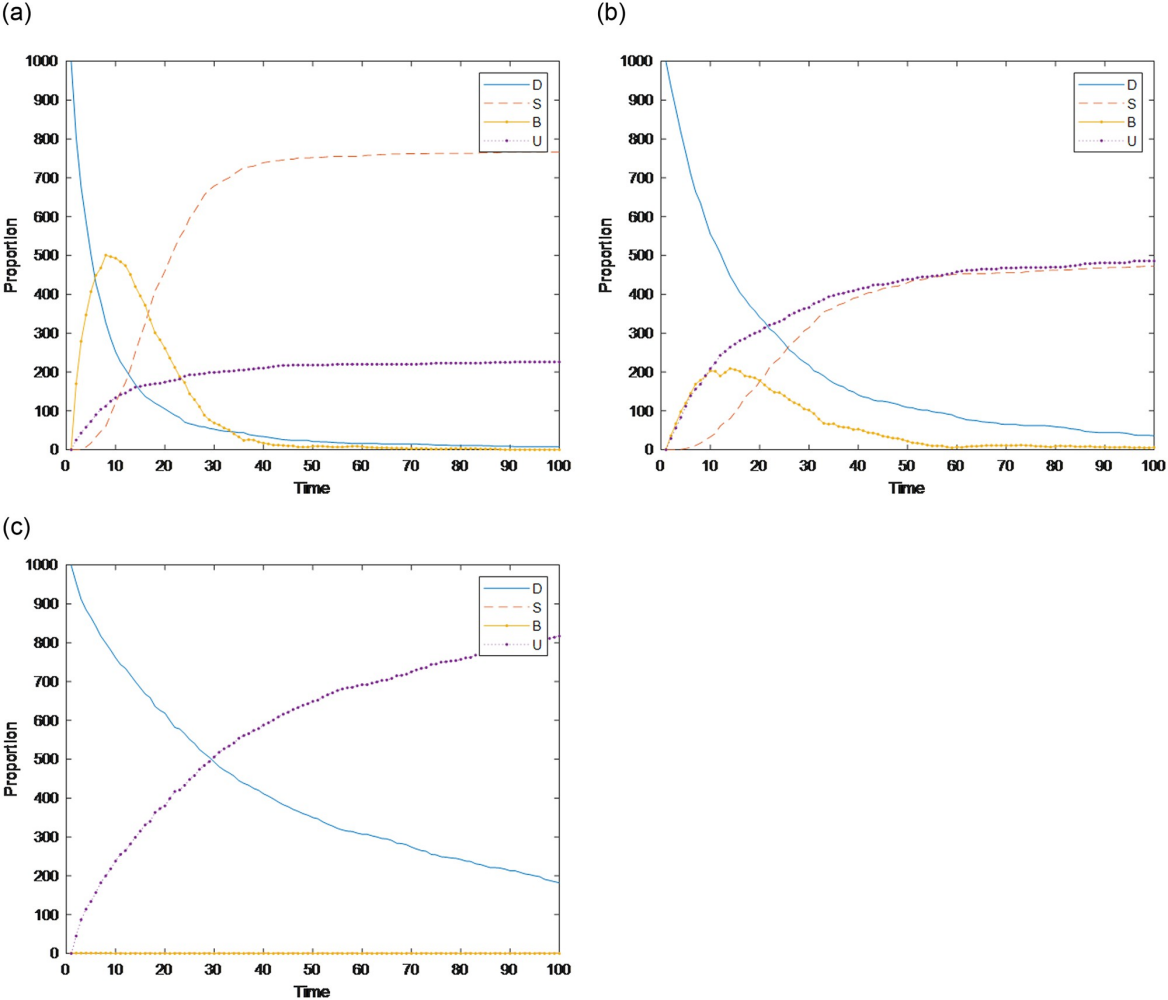
The number of burners under different network structures over time. (a) BA scale-free network, (b) WS small-world network, (c) All-connected network.

The three different networks shown in Table 4 are selected, and their impact on public opinion communication is analyzed. The results are shown in Fig 21. Fig 21(a) shows that in the process of public opinion communication in the BA scale-free network, the number of burners reaches a peak of 501 at Time = 8 and decreases to 0 at Time = 88. Fig 21(b) shows that in the WS small world network, the number of burners reaches a peak of 204 at Time = 14 and decreases to 0 for times greater than 100. Compared with the former, the time for a burner to reach its peak value and drop to 0 increases significantly with a lower peak, i.e., the communication and dissipation speed of public opinion decrease, and its communication range narrows. Fig 21(c) shows that in the all-connected network, there are almost no burners, indicating that the all-connected network restrains public opinion communication. In summary, the network structure impacts public opinion; i.e., the WS small-world network and the all-connected network restrain public opinion communication, and the latter has a strong restraining effect, while the BA scale-free network promotes public opinion communication. On the BA scale-free network, public opinion spreads faster and wider, and at the same time, it dissipates faster. The reason lies in the role of the preferred connection mechanism of the BA scale-free network; i.e., the newly added nodes are more inclined to connect with nodes with a relatively high degree in the network. A high degree of communication behavior will strongly promote the communication of public opinion among nodes. In addition, in the WS small-world network, due to the high clustering coefficient, the number of neighbors connected by a single node is large, more individuals interact, and individuals can understand the information more objectively and comprehensively. Therefore, the network structure is not conducive to network public opinion communication. The interactions of individuals in the all-connected network are more frequent, which in turn clarify their understanding of the relevant information of the event, and they ultimately lose interest in the event and choose not to spread relevant information.

## Section 6: Empirical analysis

In order to ensure the typicality of the case, three principles are followed in its selection: first, the sudden occurrence; second, the heat of the event and the great influence; third, the interests of the public. After screening, the “China Eastern Airlines MU5735 crash” event is finally selected as a typical event. The “China Eastern Airlines MU5735 crash” event, which broke out on March 21, 2022, was a public opinion event with great network influence. When the China Eastern Airlines flight MU5735 was on a mission from Kunming to Guangzhou, it lost contact and crashed over Wuzhou City, Guangxi. There were 132 people on board, including 123 passengers and 9 crew members. After the plane crash, various pieces of information about the MU5735 flooded the Internet, and it was difficult to distinguish the truth. Most of the public opinion has focused on the cause of plane crashes. In this regard, netizens have put forward many hypotheses, such as mechanical failure, broken wings, human mistakes, and the design flaws of the Boeing 737 model itself. Uncontrollable communication phenomena occur frequently, and the alleviation of negative social mentality on the whole network and the concerns and responses of online public opinion have all gathered into the online public opinion of this social disaster event.

**Fig 22:**
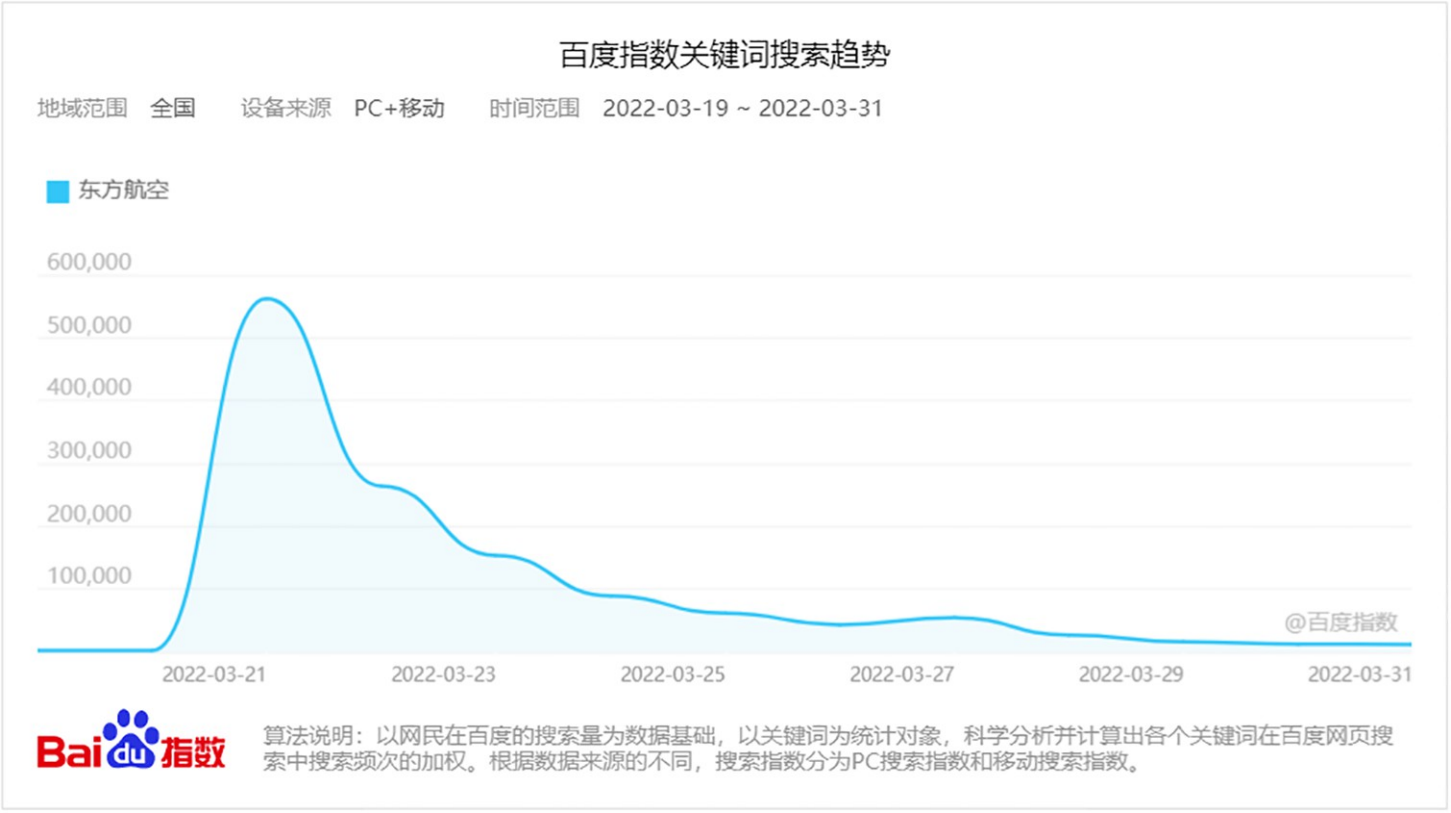
The “China Eastern Airlines MU5735 crash” event in the Baidu index.

In this research, the behavior of the corresponding key nodes in the “China Eastern Airlines MU5735 crash” event is simulated on a scale-free network, and the node size is set to 1000. Based on the DBUS communication model of network public opinion proposed above, the number of burned nodes during the period is recorded. The number of nodes and their changes, compared with the search indices from March 21 to March 31, 2022, were obtained by searching the Baidu index with “China Eastern Airlines” as the keyword. The results are shown in Fig 22 to better fit the communication process and verify the validity and rationality of the model.

**Fig 23:**
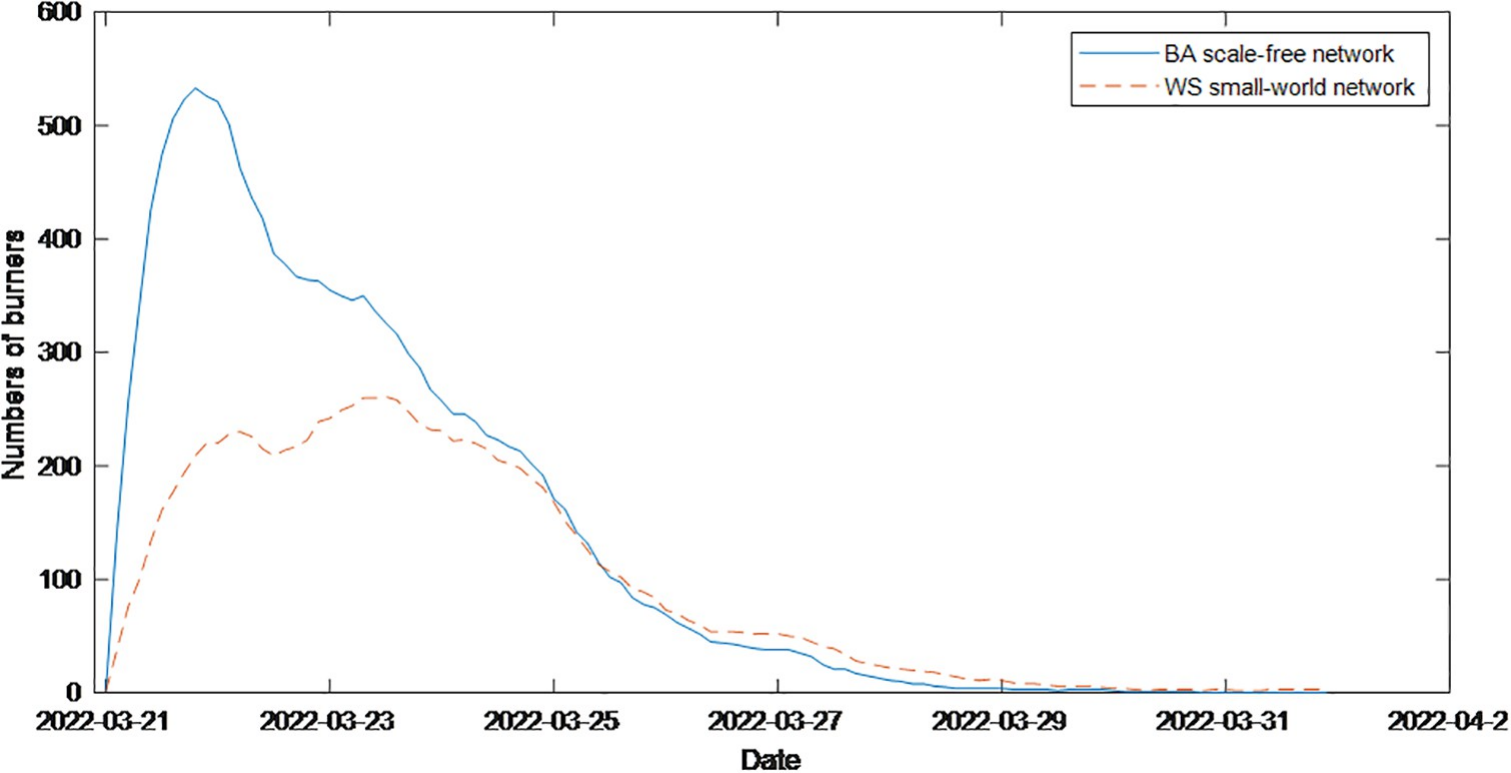
Simulation diagram of the “China Eastern MU5735 crash” event using the model proposed in this paper.

The incident lasted from March 21 to March 31. On March 21, according to Guangming.com, a China Eastern Airlines Boeing 737 passenger plane fell in Fuxian, Wuzhou City, Guangxi, and caused a wildfire. Later, according to the Civil Aviation Administration of China, it was determined that the plane had crashed. After the accident, President Xi Jinping and other state leaders immediately provided important instructions, such as activating the emergency response mechanism and making every effort to organize searches and rescues. At the same time, major media outlets reported the incident one after another, and many celebrities forwarded the news and wrote prayers. The spread of the event peaked immediately. At this time, the credibility of the government and the media ε and the transparency of the information released *θ* were set to 0.75 and 0.6 respectively. In addition, because the public did not fully understand the incident comprehensively at the time of the incident, they had constant conjectures about the cause of the accident and other issues, so various rumors and information continuously appeared. This incident involves the safety of the public’s life and is closely related to netizens. Therefore, the individual’s cognitive ability *ρ* is set to 0.65, the amount of information received by the individual is set to 0.85, and the closeness of the individual to public opinion events *b* is set to 0.9. On March 22, relevant media outlets entered the accident site for live broadcasting. The basic condition of the scene was disclosed. The National Emergency Response Command held the first press conference at 9:00 p.m. to release the search and rescue results, the cause of the accident, the search for the black box, and the rescue of family members. At this time, most netizens were rational, the individual cognitive ability *ρ* was increased to 0.9, the credibility of the government and the media *ε* was adjusted to 0.9, the transparency of information *θ* was increased to 0.8, and the amount of information received by individual *i* was adjusted to 0.7. On March 23, the black box on the crashed plane was found. In the evening, the official announced the discovery of human tissue fragments and other information at the site, which strengthened the public’s grief. At this time, the cognitive ability of netizens dropped to 0.8. On March 26, according to the paper, after 6 days of full search and rescue, the National Emergency Response Command confirmed that all the people in China Eastern Airlines flight MU5735 were dead, and the grief of the public was strengthened. The cognitive ability of netizens decreased to 0.7. On March 27, the National Emergency Response Command held mourning activities at the search and rescue site where the incident occurred. The development of the incident gradually stabilized. At this time, the cognitive ability of netizens recovered to 0.9. The simulation results are shown in Fig 23.

As shown in Fig 23, on March 21, because the truth of the incident was still unknown at the initial moment of the incident, various rumors continued to emerge, and the cognitive ability of netizens and the transparency of the released information were low. Under the influence of conformity psychology, the number of people communicating public opinion immediately reaches its peak. The next day, as the basic situation on the scene was disclosed, the cognitive ability of netizens and the role of the government and the media were strengthened, and the number of people communicating decreased significantly. On the 23rd, under the grief of the whole society, the cognitive ability of netizens decreased again, so the speed of dissipating public opinion slowed. Until the 26th, the grief strengthened again, and when the cognitive ability of netizens declined again, the speed of dissipating public opinion further slowed. On the 27th, netizens gradually accepted that all the people on board were dead, their cognitive ability returned to normal, and public opinion gradually dissipated over time. Compared with Fig 22, the number of people participating in this event peaked on the 21st, decreased rapidly on the 22nd, decreased slowly on the 23rd and 26th, and finally stabilized. Therefore, the simulation of the public opinion dissipation process during this event using the model proposed in this paper does not significantly differ from the actual results, indicating that the network public opinion communication model proposed in this paper can better simulate hot social events in reality. The model is applicable and effective and has important guiding significance for analyzing network public opinion communication.

At the same time, we are compared with the results of simulation on the WS small-world network. The simulation results are shown in Fig 23, found that consistent with the conclusion of section 5.5, public opinion spreads more slowly, has a smaller scope, and fades more slowly on WS Small World network. The trend is consistent with the reality, but there is some delay in the time nodes.

**Fig 24:**
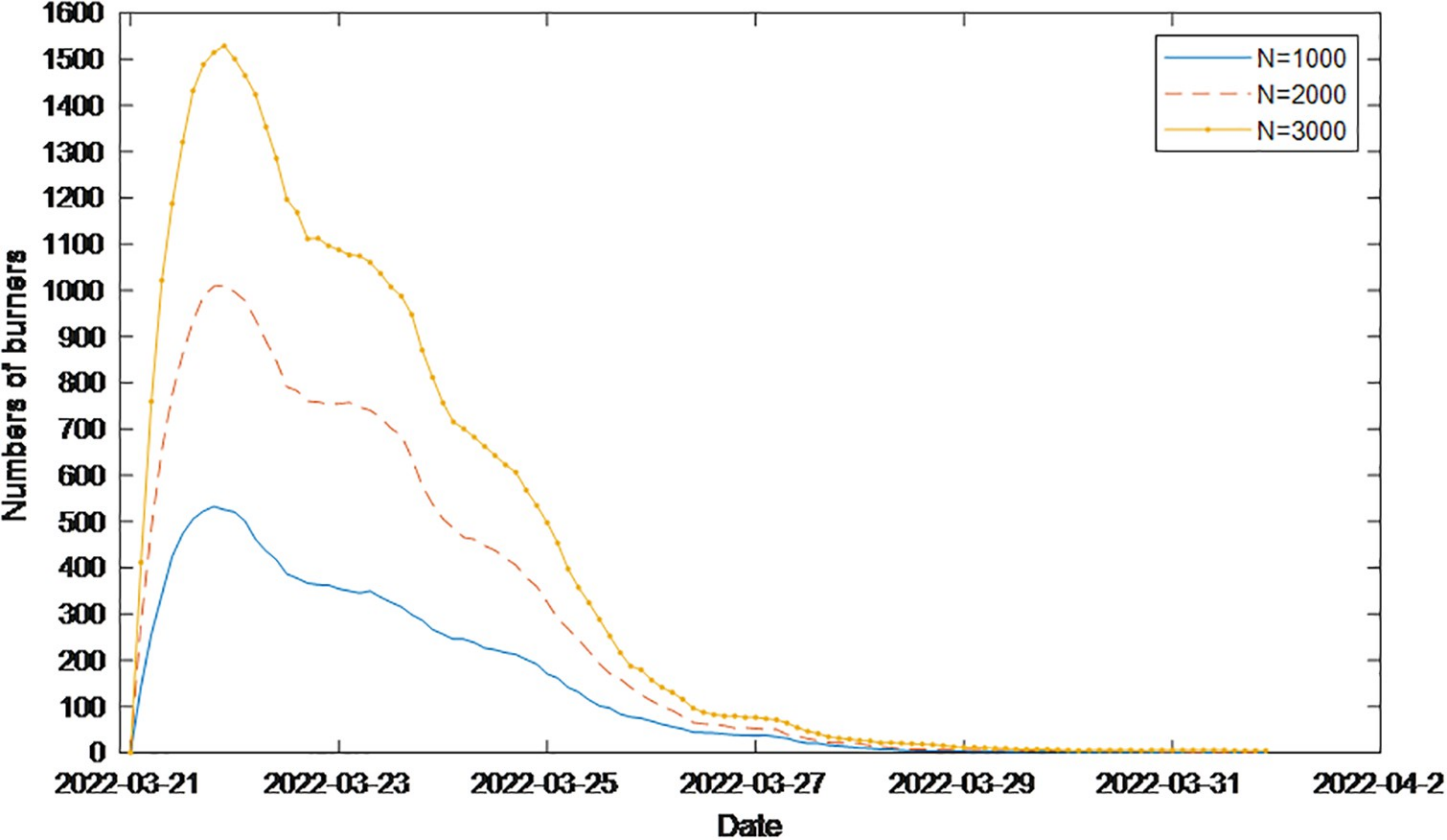
Simulation diagram of the “China Eastern MU5735 crash” event using the model proposed in this paper as nodes grow in size.

In addition, the model built in this paper is used for empirical simulation on different node sizes. The simulation results are shown in Fig 24. As shown in Fig 24, the change curve of the number of public opinion nodes in key nodes, such as the 21^st^ and 23^rd^ nodes, is consistent with the real situation, indicating that the DBUS model is still in line with the actual situation as nodes grow in size.

**Fig 25:**
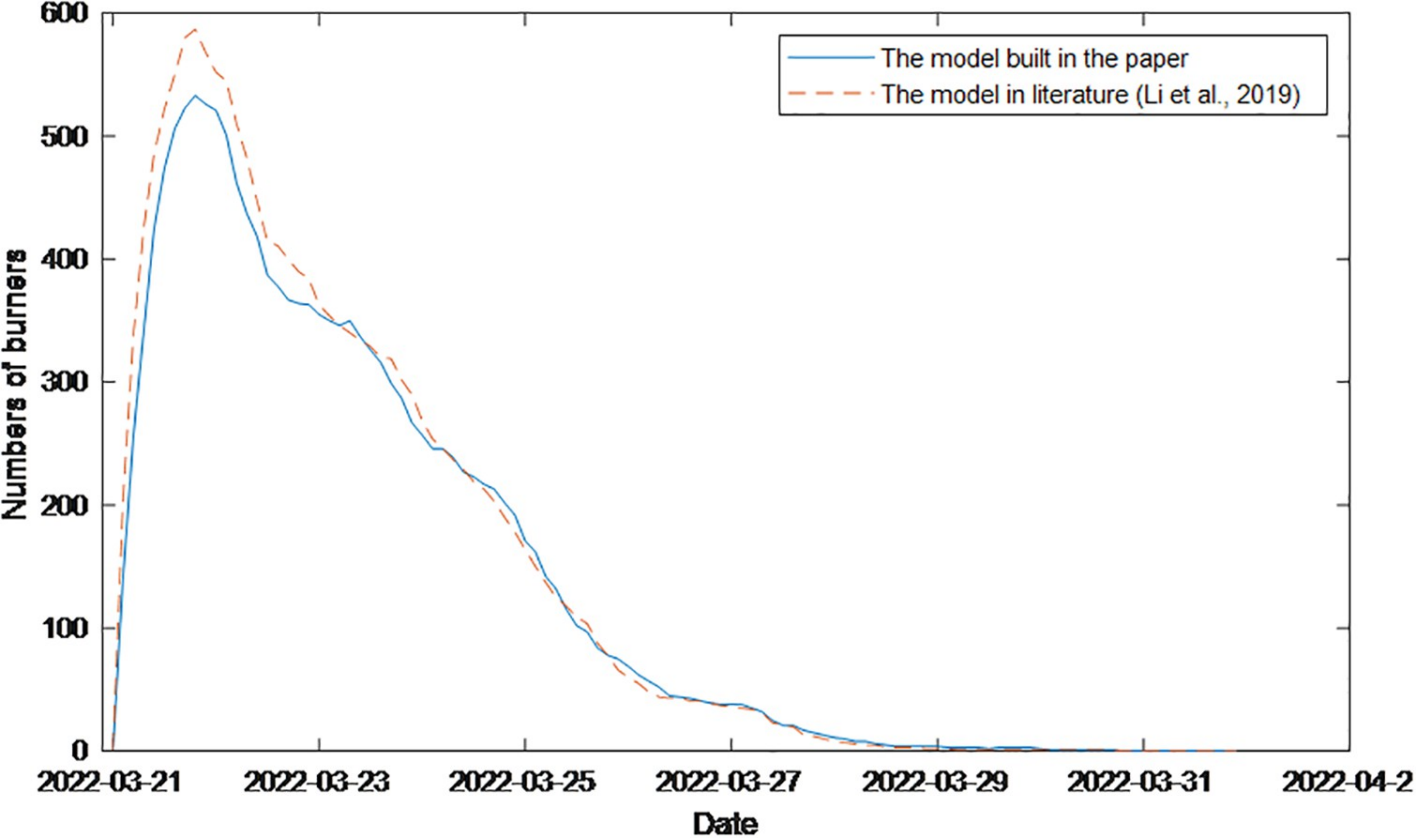
Simulation diagram of the “China Eastern MU5735 crash” event using the model in the literature [32] and the model built in this paper.

Finally, to further compare the advantages and disadvantages of the proposed models with those of other models, a model from the literature [32] is used to simulate the “China Eastern Airlines MU5735 crash” event, and the simulation results are shown in Fig 25.

Fig 25, which is the model used in the literature [32], describes the overall change trend of event communicators; however, it does not accurately describe the extent of the decline in public opinion at 23^rd^ the key time point. Fig 24 shows that there is no significant change in the reduction in the number of burners. Moreover, during the period from the 23rd to the 26th, the change curve of the communicator varies greatly, indicating that the model does not reflect the real situation of the decline in public opinion during this time period. Therefore, it can be seen from the comparison that the model proposed in this paper is more in line with the actual process of network public opinion communication.

## Section 7: Conclusions and prospects

### Section 7.1: Conclusions

This paper constructed a network public opinion DBUS communication model based on social combustion theory, and explored individuals’ cognitive ability, authority, conformity, and degree of closeness to public opinion events. Regarding the spread of information, the model is based on the amount of information received by the individual, the number of contacts between the individual and relevant public opinion information, and the influence of the role of the government and the media on public opinion communication. Finally, the feasibility and effectiveness of the model are verified through practical cases.

The following conclusions are drawn through simulation experiments:

1. Individual cognitive ability slows public opinion communication, while conformity speeds up it.
2. Individuals’ cognitive ability, authority, conformity, and role as the government and media will narrow the range of public opinion communication, while the degree of closeness of the individual to public opinion events, the number of contacts between the individual and relevant public opinion information, and the amount of information received by the individual will expand the range of public opinion communication. The credibility of the government and the transparency of the information released are the most critical factors affecting the range of public opinion communication, followed by the number of contacts, cognitive ability, and conformity.
3. Individuals’ cognitive ability, conformity, and credibility will accelerate public opinion communication, while the number of contacts inhibits the dissipation of public opinion. The individual’s cognitive ability, conformity, and the credibility of the government and the media are the key factors affecting their dissipation.
4. The WS small-world network and all-connected network can restrain public opinion communication, while the BA scale-free network can promote public opinion communication.

### Section 7.2: Limitations and prospects

This paper introduces a research framework for the social combustion theory and comprehensively considers the combined impact of individual characteristics and the external environment on the spread of online public opinion, providing a new perspective for the study of opinion dissemination and verifying its accuracy to a certain extent. However, due to the complexity of public opinion communication, the following deficiencies remain:

1. This paper does not consider the situation of derived public opinion in the process of public opinion communication. Considering that there are potential communicators in the process of public opinion communication and derivative topics after the diffusion of public opinion in the platform, the differences in the topic direction of the same public opinion will also have competition for communication, so in the subsequent research, different topic communicators can be distinguished when building the model.
2. The research in this paper is only carried out on a single layer network. In the future, we can propose the dual coupled network structure of online social network and offline social network, interpersonal social network relationship network and media network, wechat network and microblog network, and explore the communication characteristics of public opinion on this basis.
3. When the model established in this paper is applied, the specific parameters are inferred and determined according to the development of the event, and its accuracy needs to be further tested. In the future, data mining and other methods can be used to determine accurate values of these parameters.

## Supporting information

S1 File MATLAB code. (PDF)
