# Supplementary material for: Modeling of network public opinion communication based on social combustion theory under sudden social hot events
Source: PLoS One. 2024 Nov 6;19(11):e0311968. doi: 10.1371/journal.pone.0311968 (PMC11540173; doi:10.1371/journal.pone.0311968)
Supplement: S1 File — (PDF) [file pone.0311968.s001.pdf]

```
%求平均度 Average_node_size
function [N_network]=ADS(G)
d=degree(G);
N_network=sum(d)/length(d);
end

%求平均路径长度 Average_path_length
function [D_network]=APL(G)
d=distances(G);
dd=tril(d);
D_network=sum(sum(dd))/nchoosek(length(d),2);
end

function A=BA_net()
%%% 从已有的 m0 个节点的网络开始，采用增长机制与优先连接的机制生成 BA 无标度网络
%% A ——————返回生成网络的邻接矩阵
m0=input('未增长前的网络节点个数 m0:   ');
m=input('每次引入的新节点时新生成的边数 m:    ');
N=input('增长后的网络总节点数 N:    ');
disp('初始网络时 m0 个节点的连接情况：1 表示都是孤立；2 表示构成完全图；3 表示随机连接一些边');
pp=input('初始网络情况 1，2 或 3:    ');
if m>m0
    disp('输入参数 m 不合法');
    return;
end
x=100*rand(1,m0);
y=100*rand(1,m0);

switch  pp
    case 1
        A=zeros(m0);
    case 2
        A=ones(m0);
        for i=1:m0
            A(i,i)=0;
        end
    case 3
        for i=1:m0
            for j=i+1:m0
                p1=rand(1,1);
                if p1>0.5
                    A(i,j)=1;A(j,i)=0;
                end
```

```
                end
            end
        otherwise
            disp('输入参数 pp 不合法');
            return;
end

for k=m0+1:N
        M=size(A,1);
        p=zeros(1,M);
        x0=100*rand(1,1);y0=100*rand(1,1);
        x(k)=x0;y(k)=y0;
        if length(find(A==1))==0
            p(:)=1/M;
        else
            for i=1:M
                p(i)=length(find(A(i,:)==1))/length(find(A==1));
            end
        end
        pp=cumsum(p);                  %求累计概率
        for  i=1:m                         %利用赌轮法从已有的节点中随机选择  m  个节点与新加入的
节点相连
            random_data=rand(1,1);
            aa=find(pp>=random_data);jj=aa(1); %  节点 jj 即为用赌轮法选择的节点
            A(k,jj)=1;A(jj,k)=1;
        end
end
end


%求聚类系数 Clustering_coefficient
function [C_network]=Cluster(G)
C_1=adjacency(G);
a=full(C_1);
n=length(a);
for i=1:n
        m=find(a(i,:));
        ta=a(m,m);
        Lta=tril(ta);
        if length(m)==0 || length(m)==1
            c(i)=0;
        else
            c(i)=sum(sum(Lta))/nchoosek(length(m),2);
        end
```

```
end
C_network=mean(c);
end

function [G] = ERmodel
N_ER = input('网络总节点数：  '); %
p_ER =input('连接概率：  ') ; %点与点之间以 p 的概率形成连边
position=zeros(N_ER,2);      %点位置信息 position,一共有 N 组数据，每组数据有 2 个信息
adj = zeros(N_ER,N_ER);   %创建邻接矩阵，初始化邻接矩阵全零
for m=1:N_ER                   %给每个点安排位置，围成一个圆
    position(m,1)=cos(m/N_ER*2*pi);
    position(m,2)=sin(m/N_ER*2*pi);
end
figure('name','ER 随机图');
for m=1:N_ER
    for n=m+1:N_ER
        if(rand(1,1)<p_ER)   %以 0.1 的概率生成边
            adj(m,n)=1;   %这里两句给邻接表赋值
            adj(n,m)=1;
        end
    end
end
G=graph(adj,'upper');
p=plot(G,'-k','Layout','force','EdgeAlpha',0.2);
p.NodeColor = 'b';
p.MarkerSize =5;
end

%寻找周围的 B 燃烧者
function [h] = find_NB(G1,Nodes,node)
n_node=neighbors(G1,node);
h=0;
for i=1:length(n_node)
    if(Nodes(n_node(i))==2)
        h=h+1;
    end
end
end

%寻找周围的 F 抑燃者
function [h] = find_NF(G1,Nodes,node)
n_node=neighbors(G1,node);
h=0;
for i=1:length(n_node)
```

```
        if(Nodes(n_node(i))==4)
                h=h+1;
        end
end
end

%寻找周围的 S 稳定者
function [h] = find_NS(G1,Nodes,node)
n_node=neighbors(G1,node);
h=0;
for i=1:length(n_node)
        if(Nodes(n_node(i))==3)
                h=h+1;
        end
end
end

%计算燃烧者 B 的持续时间
function T_B=func1(G1,T_B)
    Nodes=table2array(G1.Nodes);
    NodeWeight=Nodes;
    for i=1:length(NodeWeight)
            if NodeWeight(i)==2
                    T_B(i)=T_B(i)+1;
            end
    end
    T_B;
end
%求燃烧者数量到达峰值的时间、燃烧者数量峰值、燃烧者数量为零的时间
function [Time_max,max_num,Time_zero]=func2(T_Nr)
T_B=T_Nr';
[max_num,Time_max]=max(T_B);
if(min(T_B)==0)
        a=find(T_B==0);
        Time_zero=a(1);
else

end
end

function [G1,g] = graghColor_withWeight(NodeWeight,Edges,g,n,N,T)
NodeTable=table(NodeWeight);
EndNodes=Edges(:,1:2);
Weight=Edges(:,3);
```

```
EdgeTable=table(EndNodes,Weight);
G1=graph(EdgeTable,NodeTable,'omitselfloops');
if n==1||n==round(T/5)||n==T
            g=g+1;
            subplot(2,2,g);
            p1=plot(G1,'-k','Layout','force');
            p1.NodeCData=G1.Nodes.NodeWeight;     %蓝 1 为感染人群，绿 2 为恢复人群，
黄 3 为易感人群,4 为另一个人群
            p1.MarkerSize =5;
            set(gca,'CLim',[1,4]); %设置颜色图上下限为 4.5、0.5
            map=[0.12 0.56 1;1 0.5 0;0 1 0;0.64 0.13 0.94]; %设计四类人员散点的颜色
            colormap(map) %更新所映射的颜色矩阵为 map
            colorbar
            text(5.2,4,['传播时间=',num2str(n)]);
            text(5.2,2,['D 阴燃者=:',num2str(N.N_infected)]);
            text(5.2,1,['S 稳定者=:',num2str(N.N_noninfected)]);
            text(5.2,0,['B 燃烧者=:',num2str(N.N_recovered)]);
            text(5.2,3,['F 抑燃者=:',num2str(N.N_E)]);
      end
end

function magnify(f1)
%
%magnify(f1)
%
%   Figure creates a magnification box when under the mouse
%   position when a button is pressed.   Press '+'/'-' while
%   button pressed to increase/decrease magnification. Press
%   '>'/'<' while button pressed to increase/decrease box size.
%   Hold 'Ctrl' while clicking to leave magnification on figure.
%
%   Example:
%        plot(1:100,randn(1,100),(1:300)/3,rand(1,300)), grid on,
%        magnify;

% Rick Hindman - 7/29/04

if (nargin == 0), f1 = gcf; end;
set(f1, ...
    'WindowButtonDownFcn',   @ButtonDownCallback, ...
    'WindowButtonUpFcn', @ButtonUpCallback, ...
    'WindowButtonMotionFcn', @ButtonMotionCallback, ...
    'KeyPressFcn', @KeyPressCallback);
return;
```

```
function ButtonDownCallback(src,eventdata)
    f1 = src;
    a1 = get(f1,'CurrentAxes');
    a2 = copyobj(a1,f1);

    set(f1, ...
        'UserData',[f1,a1,a2], ...
        'Pointer','fullcrosshair', ...
        'CurrentAxes',a2);
    set(a2, ...
        'UserData',[2,0.2], ...   %magnification, frame size
        'Color',get(a1,'Color'), ...
        'Box','on');
    xlabel(''); ylabel(''); zlabel(''); title('');
    set(get(a2,'Children'), ...
        'LineWidth', 2);
    set(a1, ...
        'Color',get(a1,'Color')*0.95);
    set(f1, ...
        'CurrentAxes',a1);
    ButtonMotionCallback(src);
return;

function ButtonUpCallback(src,eventdata)
    H = get(src,'UserData');
    f1 = H(1); a1 = H(2); a2 = H(3);
    set(a1, ...
        'Color',get(a2,'Color'));
    set(f1, ...
        'UserData',[], ...
        'Pointer','arrow', ...
        'CurrentAxes',a1);
    if ~strcmp(get(f1,'SelectionType'),'alt'),
        delete(a2);
    end;
return;

function ButtonMotionCallback(src,eventdata)
    H = get(src,'UserData');
    if ~isempty(H)
        f1 = H(1); a1 = H(2); a2 = H(3);
        a2_param = get(a2,'UserData');
        f_pos = get(f1,'Position');
```

```
            a1_pos = get(a1,'Position');

            [f_cp, a1_cp] = pointer2d(f1,a1);

            set(a2,'Position',[(f_cp./f_pos(3:4)) 0 0]+a2_param(2)*a1_pos(3)*[-1 -1 2 2]);
            a2_pos = get(a2,'Position');

        set(a2,'XLim',a1_cp(1)+(1/a2_param(1))*(a2_pos(3)/a1_pos(3))*diff(get(a1,'XLim'))*[-0.5
0.5]);
        set(a2,'YLim',a1_cp(2)+(1/a2_param(1))*(a2_pos(4)/a1_pos(4))*diff(get(a1,'YLim'))*[-0.5
0.5]);
      end;
return;

function KeyPressCallback(src,eventdata)
      H = get(gcf,'UserData');
      if ~isempty(H)
            f1 = H(1); a1 = H(2); a2 = H(3);
            a2_param = get(a2,'UserData');
            if (strcmp(get(f1,'CurrentCharacter'),'+') | strcmp(get(f1,'CurrentCharacter'),'='))
                  a2_param(1) = a2_param(1)*1.2;
            elseif (strcmp(get(f1,'CurrentCharacter'),'-') | strcmp(get(f1,'CurrentCharacter'),'_'))
                  a2_param(1) = a2_param(1)/1.2;
            elseif (strcmp(get(f1,'CurrentCharacter'),'<') | strcmp(get(f1,'CurrentCharacter'),','))
                  a2_param(2) = a2_param(2)/1.2;
            elseif (strcmp(get(f1,'CurrentCharacter'),'>') | strcmp(get(f1,'CurrentCharacter'),'.'))
                  a2_param(2) = a2_param(2)*1.2;
            end;
            set(a2,'UserData',a2_param);
        ButtonMotionCallback(src);
      end;
return;



% Included for completeness (usually in own file)
function [fig_pointer_pos, axes_pointer_val] = pointer2d(fig_hndl,axes_hndl)
%
%pointer2d(fig_hndl,axes_hndl)
%
%     Returns the coordinates of the pointer (in pixels)
%     in the desired figure (fig_hndl) and the coordinates
%           in the desired axis (axes coordinates)
%
```

```
% Example:
%   figure(1),
%   hold on,
%   for i = 1:1000,
%       [figp,axp]=pointer2d;
%       plot(axp(1),axp(2),'.','EraseMode','none');
%       drawnow;
%   end;
%   hold off

% Rick Hindman - 4/18/01

if (nargin == 0), fig_hndl = gcf; axes_hndl = gca; end;
if (nargin == 1), axes_hndl = get(fig_hndl,'CurrentAxes'); end;

set(fig_hndl,'Units','pixels');

pointer_pos = get(0,'PointerLocation');     %pixels {0,0} lower left
fig_pos = get(fig_hndl,'Position');%pixels {l,b,w,h}

fig_pointer_pos = pointer_pos - fig_pos([1,2]);
set(fig_hndl,'CurrentPoint',fig_pointer_pos);

if (isempty(axes_hndl)),
    axes_pointer_val = [];
elseif (nargout == 2),
    axes_pointer_line = get(axes_hndl,'CurrentPoint');
    axes_pointer_val = sum(axes_pointer_line)/2;
end;


%%%BA 网络的生成
A=BA_net();
G=graph(A,'upper');
figure
p=plot(G,'-k','Layout','force');
p.NodeColor = 'b';
p.MarkerSize =5;

N_infected=size(NodeWeight)-size(find(NodeWeight-1));%D 阴燃者
N_infected=N_infected(1);
T_Ni(n)=N_infected;
N_noninfected=size(NodeWeight)-size(find(NodeWeight-3));%S 稳定者
N_noninfected=N_noninfected(1);
```

```
T_Nn(n)=N_noninfected;
N_recovered=size(NodeWeight)-size(find(NodeWeight-2));%B 燃烧者
N_recovered=N_recovered(1);
T_Nr(n)=N_recovered;
N_E=size(NodeWeight)-size(find(NodeWeight-4));%F 抑燃者
N_E=N_E(1);
T_Ne(n)=N_E;
N=table(N_infected,N_noninfected,N_recovered,N_E);

function [NodeWeight,Edges] =
updateNodeState3(G1,gamma,beta,n,P_network,con_network,I_network,b_network,m_1,m_2,
T_B,qw,m_m,SD)
Nodes=table2array(G1.Nodes);
Edges=table2array(G1.Edges);
Edges1=Edges(:,1:2);
gamma;
NodeWeight=Nodes;
for node=1:size(G1.Nodes,1)
    if Nodes(node) == 1 %阴燃者 D——转燃烧者 B 或者抑燃者 F
        r = rand(); %  生成一个 0 到 1 的随机数
        %%%%%%%%阴燃者 D 转燃烧者 B
        c(node)=1-P_network(node);
        if(qw==1)
            m(node)=1-(degree(G1,node)/max(degree(G1)));
        elseif(qw==0)
            m(node)=1;
        end
        h=find_NB(G1,Nodes,node);
        T_NB(node)=con_network(node)*(h+3)/degree(G1,node);
        E_NB(node)=c(node)*m(node)*T_NB(node);
%          P(node)=1+(I_network(node)-1)*exp(b_network(node)*(1-m(node)));
%          P(node)=1+(I_network(node)-1)*exp(b_network(node)*(1-m(node)));
        P(node)=1+(I_network(node)-1)*exp(b_network(node)*(1-m_m));
%          M=m_1*(1-exp(1/m_2));
        M=m_1*(1-exp(-m_2));
%           a_B(node)=(E_NB(node)+0.3)*P(node)*(1-M);%最终版
          a_B(node)=(E_NB(node))*P(node)*(1-M);%测试版
%         a_B(node)=(1-E_NB(node))*P(node)*(1-M);
        %%%%%%%%%%%%%%
        if r < a_B(node)      %
            NodeWeight(node) = 2 ;%将节点状态设置成“B”,即燃烧者
        end
        %%%%%%%%%%阴燃者 D 转抑燃者 F
        h=find_NF(G1,Nodes,node);
```

```
            T_NF(node)=con_network(node)*(h)/degree(G1,node);
            E_NF(node)=c(node)*m(node)*T_NF(node);
%               a_F(node)=(1-E_NF(node))*(1-P(node))*M;
%               a_F(node)=(E_NF(node))*(1-P(node))*M;
%               a_F(node)=(1-E_NB(node)+0.4)*(1-P(node))*M;%最终版
                a_F(node)=(1-E_NB(node))*(1-P(node))*M;%测试版
            %%%%%%%%%%%%%%
%               if (a_B(node) <=r) && (r<=(a_F(node) +a_B(node) ) )    %
           if r < a_F(node)      %
                NodeWeight(node) = 4 ;%将节点状态设置成"F",即抑染者
            end
            %%%%%%%%%%%
        end
        if Nodes(node) == 2 %燃烧者 B 转稳定者 S
            r = rand(); %  生成一个 0 到 1 的随机数
            h=find_NS(G1,Nodes,node);
%           a_S(node)=(1-exp(-n))*h/degree(G1,node);
%               a_S(node)=1-exp(-1*T_B(node)*h/degree(G1,node)-0.01);
%          a_S(node)=1-exp(-0.01*T_B(node));%最终版
          a_S(node)=1-exp(-0.01*T_B(node)/SD);
            if r < a_S(node)        %
                NodeWeight(node) = 3 ;%将节点状态设置成"S",即稳定者
            end
        end
%%%%%%%%%%%%%%%%%%%%%%%
%         elseif Nodes(node) == 3 %易感者
%               r = rand(); %  生成一个 0 到 1 的随机数
%               k = 0 ; %  计算邻居中的感染者数量
%               for i=1:size(Edges1) %  查看所有邻居状态
%                           if   ((Edges1(i,1)   ==   node&&Nodes(Edges1(i,2))==1)||(Edges1(i,2)   ==
node&&Nodes(Edges1(i,1))==1))%如果这个邻居是感染者，则 k 加 1
%                           k = k + 1;
%                     end
%               end
%               if r < 1 - (1 - beta).^k    %  易感者被感染
%                     NodeWeight(node) = 1 ;
%               end
%         elseif Nodes(node) == 4 %易感者

        end
end

% Copyright 2015 The MathWorks, Inc.
```

```
function h = WattsStrogatz(N,K,beta)
% H = WattsStrogatz(N,K,beta) returns a Watts-Strogatz model graph with N
% nodes, N*K edges, mean node degree 2*K, and rewiring probability beta.
%
% beta = 0 is a ring lattice, and beta = 1 is a random graph.

% Connect each node to its K next and previous neighbors. This constructs
% indices for a ring lattice.
s = repelem((1:N)',1,K);
t = s + repmat(1:K,N,1);
t = mod(t-1,N)+1;

% Rewire the target node of each edge with probability beta
for source=1:N
    switchEdge = rand(K, 1) < beta;
    newTargets = rand(N, 1);
    newTargets(source) = 0;
    newTargets(s(t==source)) = 0;
    newTargets(t(source, ~switchEdge)) = 0;

    [~, ind] = sort(newTargets, 'descend');
    t(source, switchEdge) = ind(1:nnz(switchEdge));
end
h = graph(s,t);
h.Edges.Weight=ones(size(h.Edges));
figure
p=plot(h,'-k','Layout','force');
% p=plot(h,'Layout','force');
p.NodeColor = 'b';
p.MarkerSize =5;
end


clear all;clc
gamma=0.01;%恢复率
beta=0.05;%传染率
g=0;%用来画图计数的
T_Ni=0;
T_Nn=0;
T_Nr=0;
T_Ne=0;
n=1;
%%%%
disp('——————————————程序开始——————————————')
```

```
disp('—--——请输入对应数字选择要创建的网络模型：——--—')
Network_kind=input('（ER 随机网络——0、全局耦合网络——1、WS 小世界网络——2、BA 网络——3）请输入：  ');
if(Network_kind==1)
        disp('———开始全局耦合网络，请输入参数———')
        N_q=input('网络总节点数：  ');
        A=ones(N_q,N_q)-eye(N_q,N_q);
        G=graph(A,'upper');
        p=plot(G,'-k','Layout','force','EdgeAlpha',0.1);
        % p=plot(G1,'-k','Layout','force');
        p.NodeColor = 'b';
        p.MarkerSize =5;
elseif(Network_kind==2)
        disp('———开始创建 WS 小世界网络，请输入参数———')
        N_ws=input('网络总节点数：  ');
        K_ws=input('邻居节点个数：  ');
        beta_ws=input('重连概率：  ');
        G=WattsStrogatz(N_ws,K_ws,beta_ws);
elseif(Network_kind==3)
        disp('———开始创建 BA 网络，请输入参数———')
        sir001;
elseif(Network_kind==0)
        disp('———开始 ER 随机网络，请输入参数———')
        [G] = ERmodel;
else
        error('输入不符合要求,请重新启动程序');
end
%%%
disp('-——————网络基本属性—————-—')
disp(['网络的总节点数：',num2str(size(G.Nodes,1))])
disp(['网络的总边数：',num2str(size(G.Edges,1))])
[D_network]=APL(G);
disp(['网络的平均路径长度：',num2str(D_network)])
[C_network]=Cluster(G);
disp(['网络的聚类系数：',num2str(C_network)])
[N_network]=ADS(G);
disp(['网络的平均度：',num2str(N_network)])
%%%
% NN=length(A);
NN=size(G.Nodes,1);
%%%%%%%%%%%%
disp('———网络创建结束，开始创建舆论传播模型，请输入参数———')
MM=input('手动设置参数请输入 1、默认参数请输入 0：  ');
if(MM==0)
```

```
    T=100;%演化时间
    P_network_n1=0.5;
    P_network_n2=0.2;
    con_network_n1=0.5;
    con_network_n2=0.2;
    I_network_n1=0.5;
    I_network_n2=0.2;
    b_network_n1=0.5;
    b_network_n2=0.2;
    qw=1;
    m_1=0.6;
    m_2=0.8;
    m_m=5;
    SD=1;
elseif(MM==1)
    T=input('请输入演化时间 T（正整数）：  ');
    P_network_n1=input('请输入个体认知能力的正态分布参数—均值：  ');
    P_network_n2=input('请输入个体认知能力的正态分布参数—标准差：  ');
    con_network_n1=input('请输入个体从众性的正态分布参数—均值：  ');
    con_network_n2=input('请输入个体从众性的正态分布参数—标准差：  ');
    I_network_n1=input('请输入个体接受到有关舆情事件的信息量的正态分布参数—均值：
');
    I_network_n2=input('请输入个体接受到有关舆情事件的信息量的正态分布参数—标准
差：  ');
    b_network_n1=input('请输入个体信息与受众网民之间的关联程度的正态分布参数—均
值：  ');
    b_network_n2=input('请输入个体信息与受众网民之间的关联程度的正态分布参数—标
准差：  ');
    qw=input('是否考虑权威性（考虑输入 1、不考虑输入 0）：  ');
    m_1=input('请输入政府和媒体发布信息的透明度：  ');
    m_2=input('请输入政府和媒体的可信度：  ');
    m_m=input('请输入接收有关舆情信息的次数：  ');
    SD=input('请输入熄灭率下降的速度（正整数）：  ');
end
%%%%%%%%%%%%%
pause(2);
disp('———舆论传播模型创建结束———');
n_excel=input('是否需要生成数据的 excel 文件（需要输入 1、不需要输入 0）：  ');
%生成每个个体的基础属性
P_network=normrnd(P_network_n1,P_network_n2,NN,1);
con_network=normrnd(con_network_n1,con_network_n2,NN,1);
I_network=normrnd(I_network_n1,I_network_n2,NN,1);
b_network=normrnd(b_network_n1,b_network_n2,NN,1);
%剔除数值中不合理的部分，即控制范围在 0-1
```

```
while(length(find(P_network<=0))>0)||(length(find(P_network>1))>0)
        P_network(find(P_network<=0))=normrnd(0.2,0.2,length(find(P_network<=0)),1);
        P_network(find(P_network>1))=normrnd(0.2,0.2,length(find(P_network>1)),1);
end
while(length(find(con_network<=0))>0)||(length(find(con_network>1))>0)
        con_network(find(con_network<=0))=normrnd(0.2,0.2,length(find(con_network<=0)),1);
        con_network(find(con_network>1))=normrnd(0.2,0.2,length(find(con_network>1)),1);
end
while(length(find(I_network<=0))>0)||(length(find(I_network>1))>0)
        I_network(find(I_network<=0))=normrnd(0.2,0.2,length(find(I_network<=0)),1);
        I_network(find(I_network>1))=normrnd(0.2,0.2,length(find(I_network>1)),1);
end
while(length(find(b_network<=0))>0)||(length(find(b_network>1))>0)
        b_network(find(b_network<=0))=normrnd(0.2,0.2,length(find(b_network<=0)),1);
        b_network(find(b_network>1))=normrnd(0.2,0.2,length(find(b_network>1)),1);
end
NodeWeight=ones(NN,1);
NodeWeight(1)=2;
unit_statistics;
Edges=table2array(G.Edges);
figure
[G1,g]=graghColor_withWeight(NodeWeight,Edges,g,n,N,T);
T_B=zeros(NN,1);%用以储存燃烧者 B 的持续时间
for n=2:T
        T_B=func1(G1,T_B);%用以储存燃烧者 B 的持续时间

[NodeWeight,Edges]=updateNodeState3(G1,gamma,beta,n,P_network,con_network,I_network,b_network,m_1,m_2,T_B,qw,m_m,SD);%
        unit_statistics;
        [G1,g]=graghColor_withWeight(NodeWeight,Edges,g,n,N,T);
end
subplot(2,2,4);
plot([1:T],T_Ni,'-',[1:T],T_Nn,'--',[1:T],T_Nr,'.-',[1:T],T_Ne,':.');
xlabel('Time');
ylabel('Proortion');
legend('D 阴燃者','S 稳定者','B 燃烧者','F 抑燃者');
%%%%%%%%%%%%%%%%%%%%%%%%%%%%%%%%%%%%%%%%%%%%%%
figure
plot([1:T],T_Ni,'-',[1:T],T_Nn,'--',[1:T],T_Nr,'.-',[1:T],T_Ne,':.');
xlabel('Time');
ylabel('Proportion');
legend('D','S','B','U');
%%%%%%%%%%%%%%%%%%%%%%%%%%%%%%%%%%%%%%%%%%%%%%%%%%%%%%
%求燃烧者数量到达峰值的时间、燃烧者数量峰值、燃烧者数量为零的时间
```

```
[Time_max,max_num,Time_zero]=func2(T_Nr);
disp('-————————燃烧者基本属性——————-—')
disp(['燃烧者数量到达峰值的时间：',num2str(Time_max)]);
disp(['燃烧者数量峰值：',num2str(max_num)]);
disp(['燃烧者数量为零的时间：',num2str(Time_zero)]);
if (n_excel==1)
    T_D=T_Ni';T_S=T_Nn';T_B=T_Nr';T_F=T_Ne';
    T_O=ones(length(T_D),1);
    for b=1:length(T_D)
        T_O(b)=b;
    end
    xlswrite('数据.xlsx',T_O,'sheet1','A2');
    xlswrite('数据.xlsx',T_D,'sheet1','B2');
    xlswrite('数据.xlsx',T_B,'sheet1','C2');
    xlswrite('数据.xlsx',T_S,'sheet1','D2');
    xlswrite('数据.xlsx',T_F,'sheet1','E2');
    %%
    xlswrite('数据.xlsx',size(G.Nodes,1),'sheet1','G2');
    xlswrite('数据.xlsx',size(G.Edges,1),'sheet1','H2');
    xlswrite('数据.xlsx',D_network,'sheet1','I2');
    xlswrite('数据.xlsx',C_network,'sheet1','J2');
    xlswrite('数据.xlsx',N_network,'sheet1','K2');
    %%
    xlswrite('数据.xlsx',Time_max,'sheet1','G5');
    xlswrite('数据.xlsx',max_num,'sheet1','H5');
    xlswrite('数据.xlsx',Time_zero,'sheet1','I5');
    %%
    rowName=cell(1,13);
    rowName{1,1}='序号/时间';
    rowName{1,2}='D';
    rowName{1,3}='B';
    rowName{1,4}='S';
    rowName{1,5}='F';
    rowName{1,6}='总节点数';
    rowName{1,7}='总边数';
    rowName{1,8}='平均路径长度';
    rowName{1,9}='聚类系数';
    rowName{1,10}='平均度';
    rowName{1,11}='到峰值的时间';
    rowName{1,12}='峰值';
    rowName{1,13}='到零的时间';
    xlswrite('数据.xlsx',rowName(1,1),'sheet1','A1');
    xlswrite('数据.xlsx',rowName(1,2),'sheet1','B1');
    xlswrite('数据.xlsx',rowName(1,3),'sheet1','C1');
```

```
        xlswrite('数据.xlsx',rowName(1,4),'sheet1','D1');
        xlswrite('数据.xlsx',rowName(1,5),'sheet1','E1');
        xlswrite('数据.xlsx',rowName(1,6),'sheet1','G1');
        xlswrite('数据.xlsx',rowName(1,7),'sheet1','H1');
        xlswrite('数据.xlsx',rowName(1,8),'sheet1','I1');
        xlswrite('数据.xlsx',rowName(1,9),'sheet1','J1');
        xlswrite('数据.xlsx',rowName(1,10),'sheet1','K1');
        xlswrite('数据.xlsx',rowName(1,11),'sheet1','G4');
        xlswrite('数据.xlsx',rowName(1,12),'sheet1','H4');
        xlswrite('数据.xlsx',rowName(1,13),'sheet1','I4');
        %%%
        disp('———数据 excel 文件已生成，程序结束———');
elseif(n_excel==0)
        disp('—————程序结束—————');
end


clear all;
T=110;
exam=xlsread('D:\应用软件\BA 无标度网络.xlsx');
%x=1:1:100;%x 轴上的数据，第一个值代表数据开始，第二个值代表间隔，第三个值代表终止
x=exam(1:T,3);
%y=exam(1:T,5);
plot([1:T],x);
% axis([0,T,0,700]);
xlim([0,121]);
xticks(1:20:121)
set(gca,'xticklabel',{'2022-03-21','2022-03-23','2022-03-25','2022-03-27','2022-03-
29','2022-03-31','2022-04-2'});
set(gca,'YTick',[0:100:1000]);
xlabel('Date');
ylabel('Numbers of burners');
%legend('The model built in the paper','The model in literature (Li et al., 2019)');
```
